# Genomics-Informed Monitoring and Management of Genetic Diversity in a Small and Endangered Livestock Population: A Case Study of the Slovak Askanian Merino Sheep

**DOI:** 10.3390/ani16213413

**Published:** 2026-10-29

**Authors:** Adrián Halvoník, Nina Moravčíková, Ivan Pavlík, Ján Tomka, Luboš Vostrý, Hana Vostrá-Vydrová, Radovan Kasarda

**Affiliations:** 1 Institute of Nutrition and Genomics, Faculty of Agrobiology and Food Resources, Slovak University of Agriculture in Nitra, Tr. A. Hlinku 2, 94976 Nitra, Slovakiaradovan.kasarda@uniag.sk (R.K.); 2 Department of Animal Production, National Agricultural and Food Centre, Hlohovecká 2, 95141 Nitra, Slovakia; 3 Department of Genetics and Breeding, Faculty of Agrobiology, Food and Natural Resources, Czech University of Life Sciences Prague, Kamýcká 129, 16500 Prague, Czech Republic; 4 Department of Ethology and Companion Animal Science, Faculty of Agrobiology, Food and Natural Resources, Czech University of Life Sciences Prague, Kamýcká 129, 16500 Prague, Czech Republic

**Keywords:** inbreeding, effective population size, runs of homozygosity, homozygous-by-descent, mating allocation, conservation breeding

## Abstract

The Slovak Askanian Merino is a small, endangered sheep population maintained on two farms in Slovakia. This study assessed its genomic diversity, population structure and the value of genomics-informed management. After quality control, 491 individuals and 45,695 SNPs were retained for within-population analyses, while 927 individuals and 18,143 SNPs were used for comparisons with other Merino and Merino-derived breeds. Observed and expected heterozygosity were 0.411 and 0.412, respectively, while genomic inbreeding based on homozygous-by-descent segments and runs of homozygosity was 0.025 and 0.019. Segments longer than 16 Mb contributed most to inbreeding, indicating recent inbreeding. Effective population size estimates ranged from 46 to 112. Population structure analyses separated Slovak Askanian Merino from other Merino-derived breeds and revealed farm-related subdivision. Forward simulations showed that optimized mating allocation slowed inbreeding accumulation over ten generations. By generation 10, optimized scenarios reached genomic inbreeding values of 0.042–0.044 based on homozygous-by-descent segments longer than 4 Mb, compared with 0.069–0.072 under random mating. Restricting each used ram to 15–50 ewes provided the best compromise between reducing inbreeding and practical feasibility. These findings show that genomic monitoring combined with optimized mating can support the long-term conservation and sustainable management of small endangered livestock populations.

## Simple Summary

Local livestock breeds often have unique genetic characteristics shaped by generations of adaptation to local conditions, making them valuable parts of agricultural heritage. However, their small populations can quickly lose genetic diversity, threatening their long-term sustainability. The Slovak Askanian Merino is an endangered sheep population currently kept on only two farms in Slovakia. This study examined how much genetic diversity remains in the population, how the two farms are related, and whether genomic information could help improve future breeding decisions. The results show clear signs of recent inbreeding and an ongoing loss of genetic diversity. The two farms also differ genetically, which may further affect the long-term stability of the population. We therefore tested breeding strategies that use genomic information to select less-related mating pairs. Simulations showed that this approach can slow the increase in inbreeding over future generations while remaining practical for farmers. These results show that genomic information can support the long-term conservation and sustainable management of endangered local livestock breeds.

## 1. Introduction

The Merino sheep represents one of the most influential fine-wool populations worldwide. Its formation is historically associated with the progressive refinement of Mediterranean wool-producing sheep, shaped by successive introductions from Italian, North African and Iberian genetic sources. By the late Middle Ages, these processes culminated in the emergence of the Spanish Merino, a population characterized by exceptional fleece quality, which was subsequently disseminated across Europe during the 15th–18th centuries. These dispersal events initiated the development of numerous Merino-derived breeds and contributed to the establishment of a global fine-wool sheep genetic complex [1,2].

During the Soviet era, many composite fine-wool breeds were developed across the vast territory of the Soviet Union by crossing Australian Merino, American Rambouillet, Merino Précoce and Merinolandschaf with local Merino types (Novocaucasian and Mazaev Merino) and coarse-wool sheep. The Askanian Merino is one such breed, established between 1925 and 1934 at Askania Nova, located in the arid steppe of Ukraine. Local Merino ewes were initially mated to American Rambouillet rams. After the Second World War, the Askanian Merino was re-established by pure breeding of the surviving flock and by grading up fine-wool ewes imported from the North Caucasus through their mating with purebred Askanian rams [3].

Throughout the 19th and early 20th centuries, Merino and Merino-derived breeds constituted a dominant component of fine-wool production across Europe and many other regions of the world, and the Askanian Merino played an important role in this expansion. Historical records indicate that its population reached considerable levels, with approximately 1.8 million individuals documented in 1980 [3]. In recent decades, global shifts in the wool industry and a sustained decline in demand for fine-wool production have led to substantial reductions in Merino populations across several European countries. As a result, many Merino breeds are now maintained within national conservation programmes [4,5], including the Askanian Merino in Slovakia. The Slovak population is currently confined to two farms and consists of only a few hundred animals, highlighting the need for comprehensive genetic monitoring and conservation management. Despite its history, the Askanian Merino has not been comprehensively assessed using modern genomic tools, and little information is available on its current levels of genetic diversity, inbreeding and relationships with other Merino-derived breeds. Moreover, for small and endangered livestock populations, genomic monitoring should not only describe the current diversity status but should also support practical breeding decisions. In such populations, the unequal use of breeding males and mating between related individuals can rapidly increase coancestry, reduce effective population size and accelerate the loss of genetic diversity [6]. Therefore, mating allocation based on pedigree or genomic relationships represents an important tool for minimizing the expected relatedness or inbreeding of future offspring while respecting practical constraints of the breeding system. Compared with pedigree-based approaches, genomic information can capture realized relationships among individuals more accurately and may improve the management of inbreeding, particularly when pedigree depth or completeness is limited [7,8]. In conservation programmes, such approaches are especially relevant when only a restricted number of breeding males is available, and mating has to be implemented under farm-level management conditions [9].

Therefore, the aim of this study was to comprehensively assess the genomic diversity of the Slovak Askanian Merino (ASM) population using SNP data, with a focus on: (i) evaluating intrapopulation genetic diversity, including inbreeding patterns and effective population size; (ii) characterizing interpopulation genetic relationships between ASM and other Merino and Merino-derived breeds; and (iii) proposing an optimized mating scheme for this small and endangered population that is applicable under current breeding conditions. The resulting information is intended to support evidence-based conservation and management of this animal genetic resource.

## 2. Materials and Methods

### 2.1. Genomic Data and Quality Control

Nasal swab samples were collected from 502 individuals (488 ewes and 14 rams) from two farms, representing the entire active population of Slovak Askanian Merino (ASM). These samples were subsequently genotyped using the GGP Ovine 50K platform (Neogen, Lincoln, NE, USA) in a commercial laboratory. In addition, publicly available OvineSNP50 BeadChip (Illumina Inc., San Diego, CA, USA) datasets representing the Merino and Merino-derived populations listed in Table 1 were used for interpopulation analyses. Two datasets were prepared and subjected to separate quality-control procedures using PLINK v1.9 [10]. Dataset for within-population analyses and a multi-population dataset for the assessment of genetic relationships and population structure.

For the dataset containing only ASM individuals, autosomal SNPs with known genomic positions, minor allele frequency higher than 0.01, and SNP call rate higher than 0.90 were retained. Individuals with a call rate below 0.90 were excluded. The final dataset used for intrapopulation analyses contained 491 individuals, of which 14 were rams, and 45,695 autosomal SNPs. For effective population size estimation, a stricter minor allele frequency threshold of 0.05 was applied, which resulted in a dataset containing 44,887 SNPs.

Prior to the construction of the multi-population dataset, genotype data generated using the GGP Ovine 50k and OvineSNP50 BeadChip platforms were harmonized to the same ovine reference assembly Oar_v4.0. Only autosomal SNPs shared across the source datasets were considered for merging. Allele coding was compared across datasets and the harmonized datasets were subsequently merged by PLINK v1.9 [10] and subjected to the same quality control as the dataset containing only ASM individuals. In addition, Hardy–Weinberg equilibrium (HWE) filtering was performed separately within each population at *p* < 1 × 10^−6^, rather than across the pooled multi-population dataset. The final comparative dataset comprised 927 individuals and 18,143 autosomal SNPs. Prior to the ADMIXTURE analysis, linkage disequilibrium (LD) pruning was performed by PLINK v1.9 [10] using a sliding window of 50 SNPs, advanced by 5 SNPs at each step, with an r^2^ threshold of 0.2. After pruning, 16,231 SNPs were retained for the analysis.

### 2.2. Diversity Status of Slovak Askanian Merino

Genetic diversity within the Slovak Askanian Merino population was evaluated using heterozygosity measures, inbreeding coefficients and effective population size estimates. Observed heterozygosity (H_o_), expected heterozygosity (H_e_) and the inbreeding coefficient based on excess of homozygosity (F_HOM_) were calculated using PLINK v1.9 [10]. Wright’s inbreeding coefficient (F_IS_) was calculated for each SNP as (E(HET) − O(HET))/E(HET), where O(HET) and E(HET) represent the observed and expected heterozygote frequencies, respectively. Group-level F_IS_ estimates were obtained as the mean across loci, with standard errors calculated from the locus-specific F_IS_ values.

Genomic inbreeding based on autozygous segments was assessed using two complementary approaches. Runs of homozygosity (ROH) were detected in PLINK v1.9 [10] using a sliding-window algorithm. ROH parameters were set according to Ceccobelli et al. [12], who investigated inbreeding patterns in Merino and Merino-derived breeds, to ensure comparability with previously published results and to reduce bias associated with different parameter settings. Homozygous-by-descent (HBD) segments were inferred using the model-based approach implemented in the RZooRoH package v0.4.1 [13,14,15]. The model included six HBD classes (R = 2, 4, 8, 16, 32 and 64) and one non-HBD class (R = 128), following Halvoník et al. [16]. Default RZooRoH settings were used for the remaining parameters. Identified HBD segments were extracted using the rohbd function. For direct comparison with ROH, the inferred HBD segments were subsequently classified according to their physical length, independently of the HBD rate class assigned by RZooRoH package v0.4.1 [13,14,15]. The inbreeding coefficients based on ROH (F_ROH_) and HBD segments (F_HBD_) were calculated as a proportion of the autosomal genome covered by ROH or HBD segments relative to the total autosomal genome length covered by SNP markers [17]. Comparisons among farm and sex groups were descriptive only and were based on group means and standard errors; no formal hypothesis testing was performed.

Three different approaches were used to estimate the current effective population size (N_eLD_). The first approach was based on the method implemented in NeEstimator v2 [18], which applies the methodological framework described by Waples and Do [19] and is based on gamete disequilibrium. The second approach was based on artificial neural networks available in the software CurrentNe2 v1.0.1 [20]. The third approach was performed using GONE2 v1.0.2 [20], which estimates effective population size through a more complex modelling approach based on the functional relationship between gamete and linkage disequilibrium and effective population size [21].

The algorithm implemented in GONE2, which applies a genetic algorithm to derive the historical series of (N_eLD_) that best minimises the sum of squared differences between observed (D^2^) values across bins and those predicted under alternative demographic trajectories [22], was also used to estimate historical effective population size. Here, (D^2^) represents the average squared correlations between alleles at two loci, weighted by their variance.

Multiple programs were used because they differ in how LD information is processed and in their underlying methodological assumptions, allowing the consistency of Ne estimates across alternative approaches to be evaluated.

### 2.3. Genetic Connectedness to Other Populations

Pairwise genetic distances among individuals were calculated based on Nei’s genetic distance (D) using the StAMPP package v1.6.3 [23]. The resulting distance matrix was visualized as a heatmap, with hierarchical clustering applied to columns to illustrate patterns of genetic similarity. Individuals were color-coded according to breed to facilitate interpretation of clustering patterns. SplitsTree v6.7.6 [24] was used to generate a Neighbor-Net inferred from the F_ST_ matrix calculated by PLINK v1.9 [10]. A pairwise genetic distance matrix (IBS) was calculated in PLINK v1.9 [10]. The distance matrix was then imported into R 4.5.2 [25] and subjected to principal coordinates analysis using classical multidimensional scaling implemented in the cmdscale function. The first two principal coordinates were plotted to visualize the genetic structure among populations. Discriminant Analysis of Principal Components (DAPC) was performed in R using the adegenet package v2.1.11 [26]. DAPC was conducted after prior dimensionality reduction by principal component analysis. The optimal number of retained principal components (110) was determined using the optim.a.score function, which evaluates the a-score to balance discriminatory power against overfitting. Final DAPC results were visualized using scatter plots of first and second discriminant functions.

Individual ancestry proportions were inferred using ADMIXTURE v1.4.0 [27] under a model-based maximum likelihood framework assuming K ancestral populations. Values of K from 1 to 20 were evaluated using 5 replicate runs for each K and 10-fold cross-validation. The most supported number of K was further assessed using StructureSelector [28] based on the estimators MedMedK, MedMeanK, MaxMedK, and MaxMeanK. Individual membership coefficients for K = 2–20 were visualized as bar plots using pong v1.5 [29] to illustrate patterns of admixture among populations.

### 2.4. Genomic Relatedness-Based Mating Optimization and Forward Simulation of Breeding Scenarios

Pairwise genomic relatedness (*r*) among individuals was derived from the identity-by-descent (IBD) matrix calculated using PLINK v1.9 [10]. PI_HAT used as *r* was calculated as Z_2_ + 0.5 Z_1_, where Z_1_ and Z_2_ represent the estimated probabilities that a pair of individuals shares one or two alleles identical by descent (IBD), respectively. The average genomic relatedness of each ram was calculated as the average *r* value across all pairwise combinations between the given ram and all individuals in the population. Self-comparisons were excluded from all pairwise relatedness calculations. The genomic diversity index (GDI) was calculated as follows:

*GDI* = *F*_ROH>16 Mb_ + *r* + *A_REF_*

where F_ROH>16Mb_ represents genomic inbreeding based on ROH longer than 16 Mb, *r* is the average genomic relatedness of an individual to the population, and A_REF_ reflects the contribution of an individual to the frequency of rare alleles. Lower GDI values indicate a more favourable contribution to population genomic diversity. For a full description of the methodology, see [30]. In the present study, GDI was used as a supporting indicator, whereas the subsequent mating optimization was based directly on genomic relatedness.

Mating allocation was formulated as a binary linear programming problem and implemented in R 4.5.2 using the lpSolve package v5.6.23 [31]. For optimized scenarios, pairwise genomic relatedness values were used to construct matrices representing all possible ewe–ram combinations allowed under the farm structure. Each potential ewe–ram combination was represented by a binary decision variable indicating whether the mating was selected. The objective of the model was to minimize the sum of pairwise genomic relatedness values across all selected ewe–ram mating pairs. Since each ewe was assigned to exactly one ram, minimizing the sum of relatedness values was equivalent to minimizing the average genomic relatedness of the proposed mating pairs. Three ram-use strategies were evaluated. In all scenarios, three of the 14 available rams were assigned to Farm 1 and the remaining 11 to Farm 2, reflecting the different numbers of breeding ewes in the two farms (104 and 373, respectively). Each ram could be assigned to only one farm within a given breeding cycle. In the balanced strategy, all available rams were used and ewes were distributed as evenly as possible among the rams assigned to each farm, resulting in 34–35 ewes per ram in Farm 1 and 33–34 ewes per ram in Farm 2. In the Max50 strategy, each used ram was assigned at least one ewe and no more than 50 ewes. In the Min15_Max50 strategy, both lower and upper limits were applied, restricting the number of ewes assigned to each used ram to between 15 and 50. In the optimized scenarios, the identities of the three rams assigned to Farm 1 and the 11 rams assigned to Farm 2, together with the individual ewe–ram combinations, were determined by the optimization algorithm to minimize total pairwise genomic relatedness. In the corresponding random scenarios, rams were randomly allocated between farms under the same 3:11 farm allocation and mating-intensity constraints, and ewes were subsequently assigned randomly to the rams available within each farm.

To evaluate the consequences of alternative mating strategies under the current breeding structure of the ASM population, a forward stochastic simulation framework was developed. Each scenario was simulated for ten generations, with generation 0 representing the observed founder population. Fifteen replicate simulations were generated per scenario and a unique seed was assigned to each replicate. Genotypes were phased by chromosome using SHAPEIT2 v2.r904 [32] and phased haplotypes from all autosomes were subsequently merged and imported into AlphaSimR v2.1.0 [33] as founder haplotypes together with the corresponding marker map. The simulated founder population represented the active breeding population of ASM. Ewes were assigned to their original farms, with 104 ewes in farm 1 and 373 ewes in farm 2, while all rams were assigned to a common sire pool. This structure reflected the practical management situation in which ewes remain farm-specific, whereas rams can be allocated to either farm. However, to represent natural mating conditions, each ram could be used on only one farm within a given mating season and could not be assigned simultaneously to both farms in one season.

To approximate the age and replacement structure of the breeding population, animals were divided into reproductive classes. Ewes were represented by four reproductive classes within each farm. In farm 1, classes O1, O2, O3 and O4 included 37, 28, 22 and 17 ewes, respectively. In farm 2, the corresponding class sizes were 133, 102, 78 and 60 ewes. Rams were represented by three reproductive classes, B1, B2 and B3, including 7, 4 and 3 rams, respectively. This structure defined the number of replacement animals required in each simulated breeding cycle and ensured that the total number of breeding animals remained constant across generations. Each simulated breeding cycle consisted of mating allocation, lambing, replacement selection, ageing and culling. At the beginning of each cycle, all reproductive ewes were assigned to one ram according to the scenario-specific mating rules. Litter size was then simulated independently for each mated ewe, allowing ewes to produce no lamb, one lamb or twin lambs. The probabilities of litter sizes 0, 1 and 2 were set to 0.15, 0.66 and 0.19, respectively, based on the reproductive performance reported for Ascanian Merino by Lobachova [34]. The sex of each lamb was assigned randomly with equal probability for females and males. This allowed the simulation to account for variation in fertility and litter size while keeping the same reproductive assumptions across all scenarios. Replacement animals were selected randomly from the available offspring. In each cycle, 37 female lambs born in farm 1 and 133 female lambs born in farm 2 were selected as replacement ewes and entered reproductive class O1 in the next cycle. In addition, 7 male lambs were selected from all male offspring as replacement rams and entered class B1. The remaining animals were not retained for breeding. Existing breeding animals were then aged between reproductive classes: in farm 1, 28 ewes were retained from O1 to O2, 22 from O2 to O3 and 17 from O3 to O4; in farm 2, 102 ewes were retained from O1 to O2, 78 from O2 to O3 and 60 from O3 to O4. Ewes in O4 were removed after the breeding cycle. Among rams, 4 animals were retained from B1 to B2 and 3 from B2 to B3, while B3 rams were removed. This replacement and ageing process maintained a constant population size of 477 ewes and 14 rams in every simulated generation.

To evaluate simulated mating allocations, F_HBD_ was calculated according to the methodology described in the section Diversity status of Slovak Askanian Merino. Changes in farm-based population structure were evaluated using genome-wide F_ST_ calculated in PLINK v1.9 [10]. For all evaluated parameters, results were summarized by scenario and generation, and means with standard errors were calculated across simulation replicates.

## 3. Results

### 3.1. Diversity Status of the Slovak Askanian Merino

The average H_o_ and He in the ASM population were 0.411 ± 0.001 and 0.412 ± 0.001, respectively (Table 2). Farm 2 showed higher heterozygosity than Farm 1, with H_o_ values of 0.416 ± 0.008 and 0.396 ± 0.003, respectively. The same pattern was observed for H_e_, which reached 0.412 ± 0.001 in Farm 2 and 0.369 ± 0.001 in Farm 1. Values of H_o_ were similar in rams and ewes, whereas H_e_ was lower in rams than in ewes. The average F_HOM_ for the whole ASM population was 0.004 ± 0.003. The corresponding F_IS_ value was 0.004 ± 0.000. F_HOM_ differed between farms. Farm 1 had a positive F_HOM_ value of 0.041 ± 0.008. Farm 2 had a negative F_HOM_ value of −0.008 ± 0.002. F_IS_ was negative in both farms. The values were −0.066 ± 0.001 in Farm 1 and −0.010 ± 0.000 in Farm 2. Rams also had a negative F_IS_ value of −0.144 ± 0.001.

**Figure 1:**
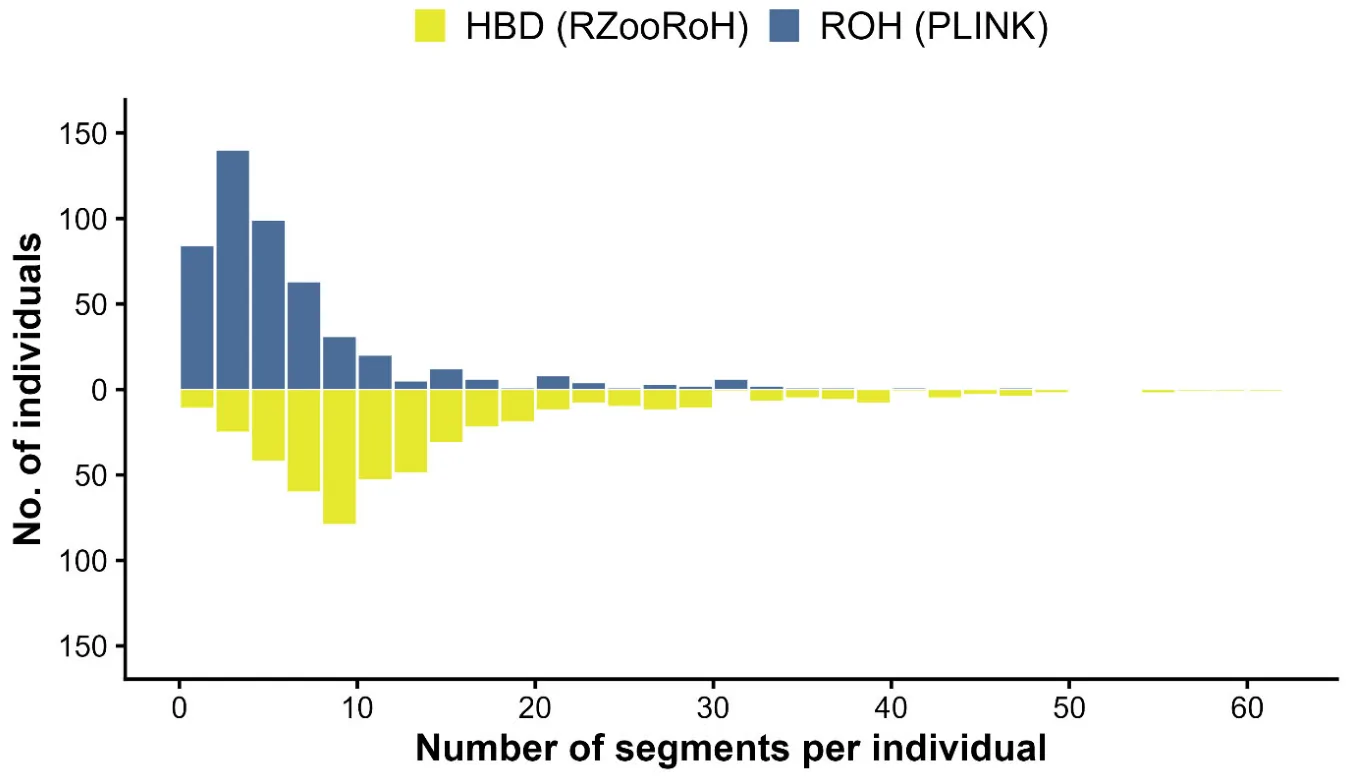
Number of detected ROH (**top**—blue) and HBD segments (**bottom**—yellow) per individual in the Slovak Askanian Merino population.

A total of 2880 ROH and 6860 HBD segments were detected in the ASM population. The average number of ROH per individual was 5.866 ± 0.301. The average number of HBD segments per individual was 13.971 ± 0.493. The higher number of HBD segments was mainly due to short segments. In the 1 to 2 Mb category, the average number was 0.648 ± 0.039 for ROH and 6.623 ± 0.227 for HBD. In the 2 to 4 Mb category, the average number was 1.768 ± 0.078 for ROH and 3.662 ± 0.133 for HBD. Differences between the two approaches were negligible in longer segments (Table 3). The number of detected autozygous segments varied among individuals and population groups (Figure 1; Supplementary Tables S1 and S2). Individual values ranged from 0 to 47 ROH and from 0 to 60 HBD segments. Farm-related differences were evident for both approaches. Farm 1 showed a higher average number of HBD segments than Farm 2 (17.195 ± 1.346 vs. 12.952 ± 0.480) and also a higher average number of ROH segments (9.237 ± 0.950 vs. 4.799 ± 0.233). The largest farm differences were observed in longer segment classes, especially in segments longer than 16 Mb. In this length class, Farm 1 showed 2.551 ± 0.403 HBD and 2.542 ± 0.403 ROH segments per individual, while Farm 2 showed 0.343 ± 0.078 HBD and 0.311 ± 0.075 ROH segments per individual. Differences between rams and ewes were less clear at the level of total segment counts, but rams showed a higher average number of segments longer than 16 Mb in both HBD and ROH analyses.

**Figure 2:**
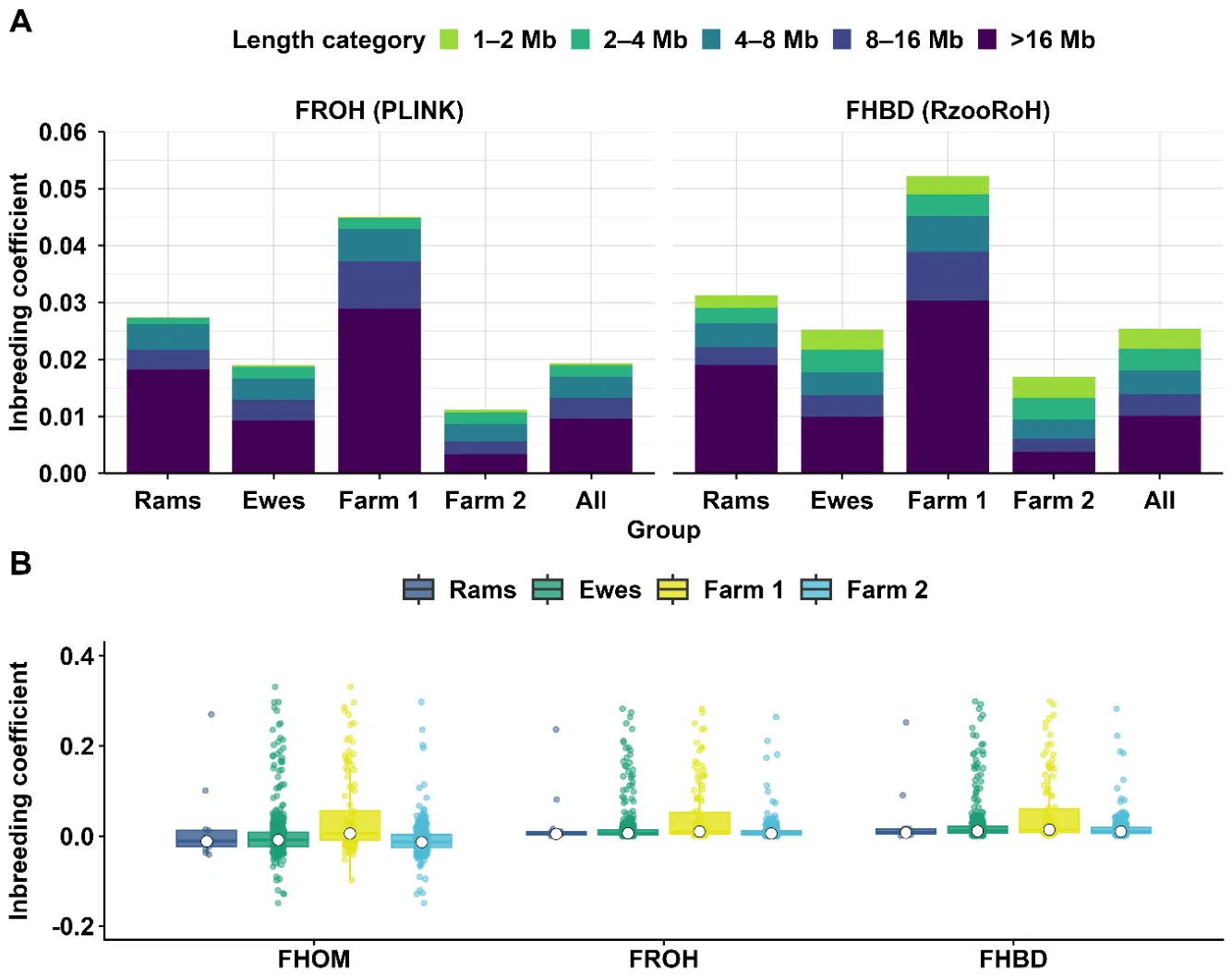
Genomic inbreeding patterns in the Slovak Askanian Merino population. (**A**)—Contribution of different ROH and HBD length categories to F_ROH_ and F_HBD_ across analysed groups. (**B**)—Distribution of individual genomic inbreeding coefficients (F_HOM_, F_ROH_ and F_HBD_) among individuals of the analysed groups. Group sizes were *N* = 14 for rams, *N* = 477 for ewes, *N* = 118 for Farm 1 and *N* = 373 for Farm 2.

The higher number of HBD segments was reflected in higher F_HBD_ relative to F_ROH_. The average F_HBD_ in the whole ASM population was 0.025 ± 0.002, while the average F_ROH_ was 0.019 ± 0.002 (Table 2). As shown in Figure 2A, values of F_HBD_ and F_ROH_ differ between farms and sexes. Farm 1 had higher F_HBD_ and F_ROH_ than Farm 2. The average F_HBD_ was 0.052 ± 0.006 in Farm 1 and 0.017 ± 0.001 in Farm 2. The corresponding F_ROH_ values were 0.045 ± 0.006 and 0.011 ± 0.001. Rams had slightly higher average F_HBD_ and F_ROH_ than ewes. In both F_HBD_ and F_ROH_, the largest contribution originated from segments longer than 16 Mb, indicating recent inbreeding. As shown in Figure 2B, individual inbreeding coefficients showed wide variation within the ASM population. Individual F_HBD_ values ranged from 0 to 0.300. Individual F_ROH_ values ranged from 0 to 0.282. F_HOM_ also varied among individuals, with values from −0.205 to 0.331.

**Figure 3:**
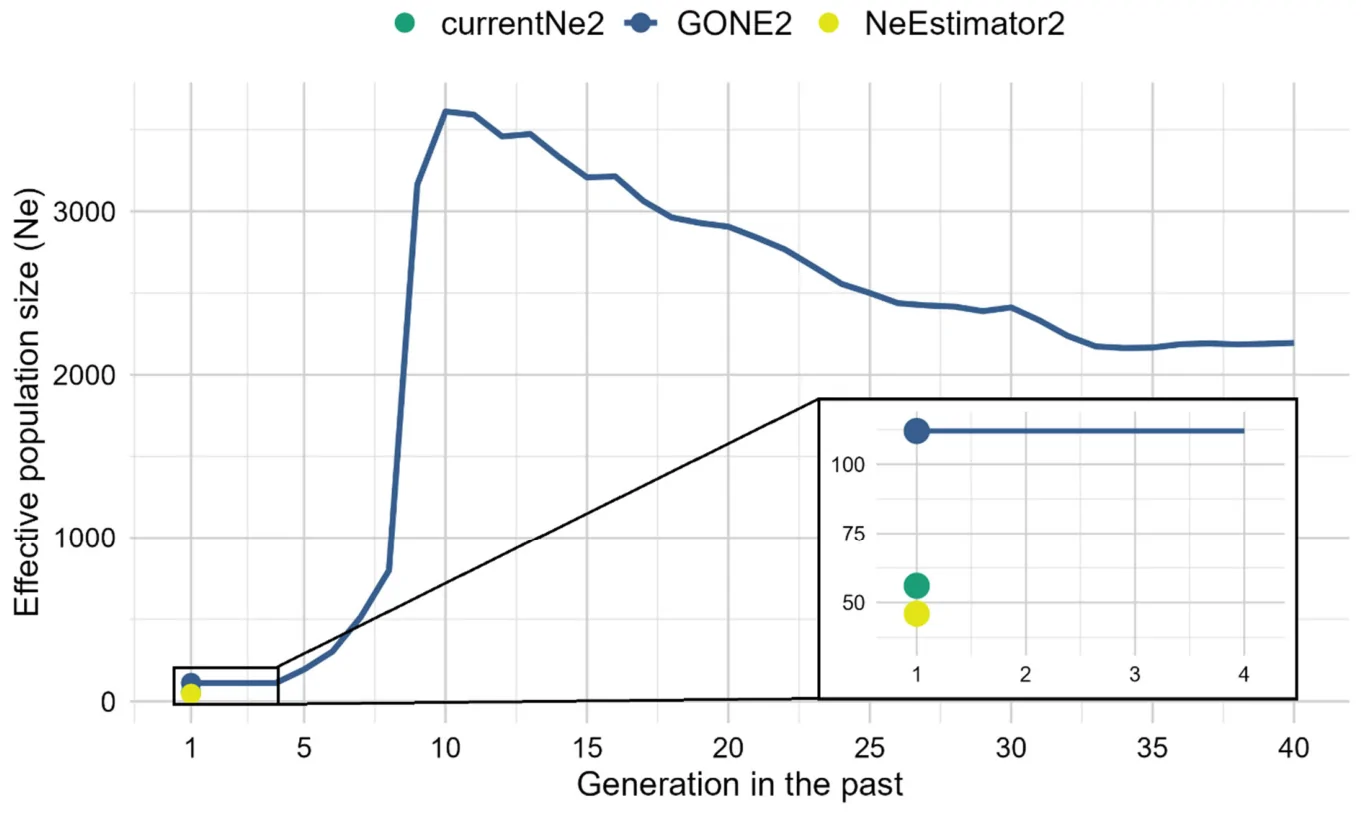
Estimation of current and historical gametic/linkage effective population size of the Slovak Askanian Merino population.

Contemporary N_e_ estimates based on linkage disequilibrium/gametic ranged from 46 to 112 individuals. NeEstimator2 produced the lowest estimate, with Ne = 46. The estimate from currentNe2 was 56. GONE2 produced the highest contemporary estimate, with Ne = 112. As shown in Figure 3 the historical N_e_ trajectory estimated by GONE2 showed a decrease toward the present. The highest estimated N_e_ was 3164 individuals approximately nine generations ago.

### 3.2. Population Structure

**Figure 4:**
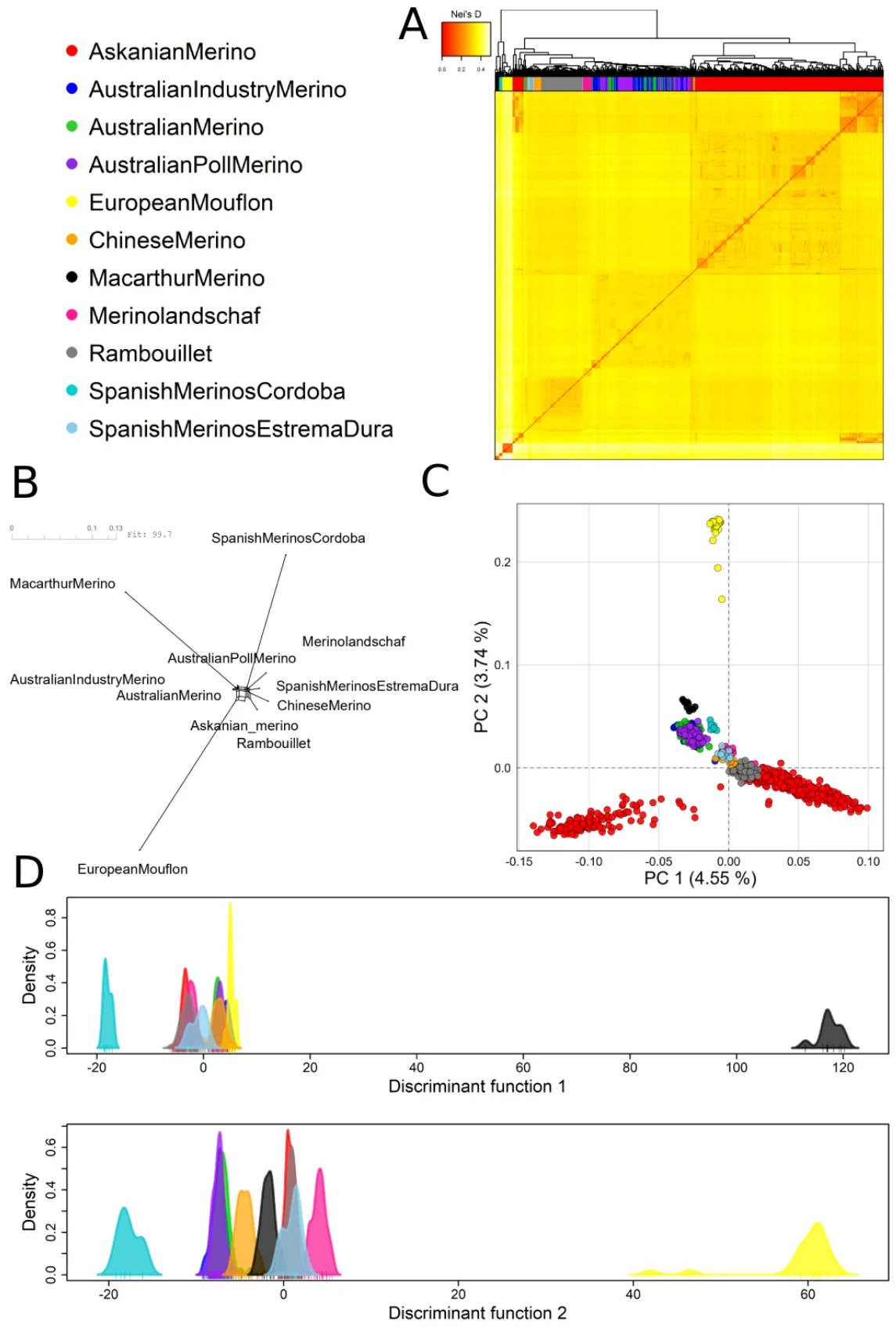
Population structure of Merino and Merino-derived breeds. (**A**)—Heatmap of individual-level Nei’s genetic distances. (**B**)—Neighbor-Net inferred from the F_ST_ matrix. (**C**)—PCoA based on the IBS distance matrix. (**D**)—First and second discriminant functions of DAPC analysis.

The heatmap based on Nei’s genetic distances (Figure 4A) showed clear clustering of individuals according to population origin. The ASM population formed a separate cluster from the other analysed Merino and Merino-derived breeds, while internal clustering corresponding to the two farms was also visible. The lowest Nei’s distances from ASM were observed for Australian Poll Merino (D = 0.029), Rambouillet (D = 0.030), Australian Industry Merino (D = 0.032) and Australian Merino (D = 0.033). Slightly higher values were found between ASM and Spanish Merinos Estrema Dura (D = 0.037) and Chinese Merino (D = 0.038). The greatest distances from ASM were observed for Spanish Merinos Cordoba (D = 0.164), Macarthur Merino (D = 0.167) and European Mouflon (D = 0.185).

Pairwise F_ST_ (Figure 4B) showed a similar pattern of genetic differentiation among populations as Nei’s genetic distances. The lowest F_ST_ values involving ASM were observed with Spanish Merinos Estrema Dura (F_ST_ = 0.028), Australian Poll Merino (F_ST_ = 0.034), Australian Merino (F_ST_ = 0.035), Australian Industry Merino (F_ST_ = 0.035), Rambouillet (F_ST_ = 0.035) and Chinese Merino (F_ST_ = 0.038). A higher level of differentiation was observed between ASM and Merinolandschaf (F_ST_ = 0.046). The highest pairwise F_ST_ values involving ASM were detected with Spanish Merinos Cordoba (F_ST_ = 0.171), Macarthur Merino (F_ST_ = 0.182) and European Mouflon (F_ST_ = 0.210).

Graphical visualisation of the first two principal coordinates (Figure 4C) separated the ASM population from other Merino and Merino-derived breeds. Moreover, the ASM population was divided into two subpopulations following the distribution of individuals between farms. Regarding the relationship between ASM and other analysed breeds, the genetically closest was Rambouillet, followed by Chinese Merino. Among the analysed breeds, the European Mouflon was most distinct from all domestic breeds, consistent with its status as a genetically distinct wild population.

The DAPC analysis (Figure 4D) provided an additional view of differentiation among the analysed populations. The first discriminant function clearly separated Macarthur Merino and Spanish Merino Cordoba, while the European Mouflon, together with other domestic breeds, formed one joint genetic cluster. The second discriminant function, unlike the first, clearly separated the European Mouflon from all other analysed breeds.

**Figure 5:**
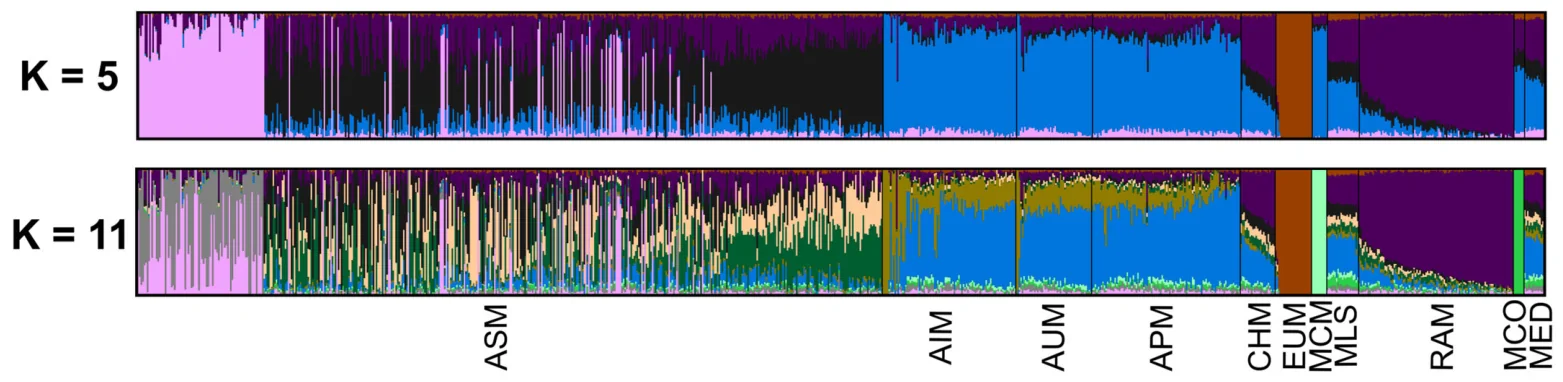
Proportion of genetic admixture among Merino and Merino-derived breeds at K = 5 and K = 11. ASM—Slovak Askanian Merino, AIM—Australian Industry Merino, AUM—Australian Merino, APM—Australian Poll Merino, CHM—Chinese Merino, EUM—European Mouflon, MCM—Macarthur Merino, MLS—Merinolandschaf, RAM—Rambouillet, MCO—Spanish Merinos Cordoba, MED—Spanish Merinos Estrema Dura.

Unsupervised clustering was used to further characterize ancestry profiles among the analysed populations (Figure 5). The StructureSelector results identified K = 5 (Supplementary Figure S1) as the best-supported clustering level, while K = 11 was additionally presented because it corresponds to the number of analysed populations. Results across the full range of K = 2–20 are presented in Supplementary Figure S2. At K = 5, the European Mouflon showed almost complete assignment to a single ancestry component (average q = 0.972), consistent with its separation from domestic sheep in the preceding analyses. Among domestic populations, Australian Industry Merino, Australian Merino, Australian Poll Merino and Macarthur Merino were mainly assigned to the same component, with average membership probabilities ranging from 0.768 to 0.880. Rambouillet was mainly assigned to a different component (average q = 0.597), whereas Chinese Merino showed a more mixed profile, with its two main components reaching average values of 0.367 and 0.337. The ASM population showed a mixed ancestry profile at K = 5 and was not assigned to a single dominant component. Across all ASM individuals, the three main components reached average membership probabilities of 0.468, 0.216 and 0.194. The farm-related structure observed in the previous analyses was also visible in the ADMIXTURE results. Farm 1 showed strong assignment to one component (average q = 0.714), whereas Farm 2 was mainly assigned to a different component (average q = 0.599), with a secondary component reaching 0.192.

At K = 11, similar overall patterns were observed. Macarthur Merino was completely assigned to a distinct component (average q = 1), and European Mouflon remained almost completely assigned to its own component (average q = 0.972). Rambouillet also showed a high average assignment to one component (average q = 0.798), while the Australian Merino-derived populations showed their highest membership in another shared component, with average q values ranging from 0.651 to 0.664. In contrast, Chinese Merino, Merinolandschaf and the two Spanish Merino populations retained more mixed ancestry profiles at K = 11. The ASM population also remained heterogeneous at K = 11. No single private component dominated across the whole ASM population, with the four largest average components reaching 0.144, 0.139, 0.136 and 0.133. However, the two farms differed markedly in their ancestry profiles. In Farm 1, three components accounted for most of the average ancestry profile, with q values of 0.395, 0.383 and 0.110. In Farm 2, the four main components reached average q values of 0.187, 0.180, 0.174 and 0.168. These results further support the presence of farm-related substructure within the Slovak ASM population.

### 3.3. Genomic Relatedness-Based Mating Optimization and Forward Simulation of Breeding Scenarios

Substantial variability in the average genomic relatedness and GDI of ASM rams was observed (Table 4), indicating differences in their potential contribution to the breeding population. Average genomic relatedness ranged from 0.034 to 0.150. Most rams showed relatively similar intermediate values, whereas one ram exhibited a markedly higher average relatedness to the population. GDI values also differed among rams, ranging from 0.045 to 0.236, suggesting that individual rams differed not only in their relatedness to the population but also in their relative value for maintaining genomic diversity.

Initial mating optimization was first evaluated at the level of selected ewe–ram pairs to compare the immediate effect of the three optimized ram-use strategies. The lowest average genomic relatedness between selected mating pairs was achieved under the Max50 strategy (0.0088), followed by the Min15_Max50 strategy (0.0095), whereas the balanced strategy resulted in the highest value (0.0113). In the balanced strategy, ewes were distributed almost evenly among rams, with 34–35 ewes per ram in farm 1 and 33–34 ewes per ram in farm 2. In contrast, the Max50 strategy allowed a more uneven allocation, with some rams assigned to only 1–4 ewes. The Min15_Max50 strategy reduced this imbalance by constraining the number of assigned ewes per ram to between 15 and 50.

**Figure 6:**
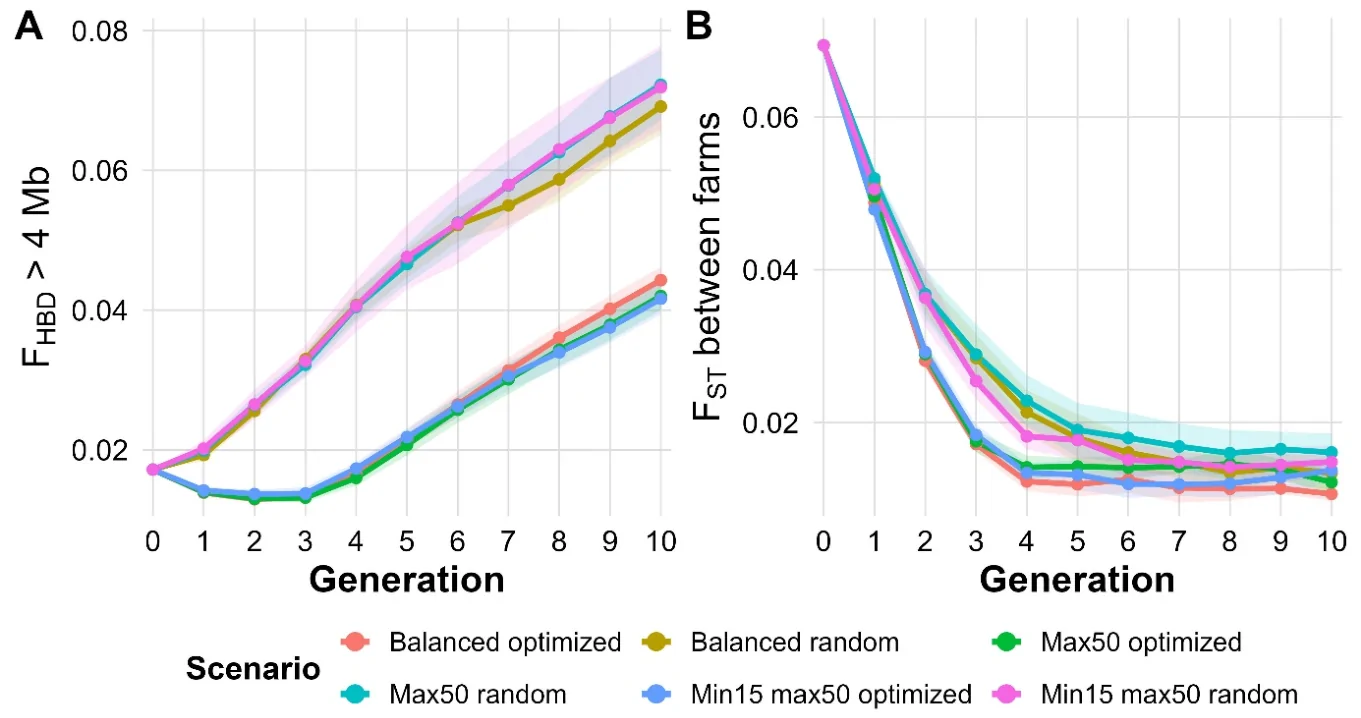
Changes in genomic inbreeding and genetic differentiation between farms across simulated mating scenarios. (**A**) Average F_HBD_ calculated from homozygous-by-descent segments longer than 4 Mb. (**B**) F_ST_ between farms. Lines represent average values across simulation replicates, and shaded areas represent 95% uncertainty intervals around the mean. Generation 0 represents the observed founder population.

Because the lowest immediate relatedness does not necessarily represent the most favourable long-term population outcome, the six mating scenarios were further evaluated using forward simulations, simulations demonstrated clear differences among scenarios in the accumulation of F_HBD>4 Mb_ and in the development of genetic differentiation between farms (Figure 6). At generation 0, all scenarios started from the same F_HBD>4 Mb_ value of 0.017. In the optimized scenarios, F_HBD>4 Mb_ initially decreased and remained below the founder level during the first three generations, reaching 0.013–0.014 by generation 3. In contrast, random mating scenarios showed a continuous increase from the beginning of the simulation. After generation 3, F_HBD>4 Mb_ increased in all scenarios, but the increase remained consistently lower under optimized mating allocation than under random mating. By generation 10, F_HBD>4 Mb_ reached 0.044 in Balanced optimized, 0.042 in Max50 optimized and 0.042 in Min15_Max50 optimized, compared with 0.069, 0.072 and 0.072 in the corresponding random scenarios. Thus, optimized mating allocation reduced the accumulation of F_HBD>4 Mb_ by approximately 36–42% compared with random mating within the same breeding structure.

A similar pattern was observed for the other F_HBD_ length thresholds, which are presented in the Supplementary Information. Across thresholds of 2 Mb, 8 Mb and 16 Mb, optimized scenarios consistently showed lower inbreeding levels than the corresponding random scenarios at the end of the simulation (Supplementary Tables S3–S6). This indicates that the effect of optimized mating was not restricted to a single F_HBD_ category but was observed across short, intermediate and long HBD segments.

The marked decline in F_ST_ observed across both random and optimized scenarios was therefore primarily associated with the use of a common sire pool. Within this shared-sire framework, the optimized scenarios generally showed slightly lower F_ST_ values than the corresponding random scenarios. By generation 10, F_ST_ reached 0.011 in Balanced optimized, 0.012 in Max50 optimized and 0.014 in Min15_Max50 optimized, compared with 0.013–0.016 in the corresponding random scenarios. Thus, genomic optimization provided an additional, but smaller, reduction in farm differentiation within the common-sire breeding structure. Detailed F_ST_ values across generations are presented in Supplementary Table S7.

## 4. Discussion

### 4.1. Diversity Status of the Slovak Askanian Merino

The ASM population exhibited higher observed and expected heterozygosity compared to other Merino and Merino-derived breeds reported by Ceccobelli et al. [12], where average values of Ho and He across populations were 0.375 and 0.380, respectively. In addition, ASM showed a lower level of F_ROH_ than the majority of Merino populations analyzed by Ceccobelli et al. [12], which could be interpreted as relatively favourable genetic diversity. However, a more detailed analysis of ROH length classes and HBD segments revealed that the largest proportion of inbreeding in the ASM population originated from segments longer than 16 Mb, reflecting recent inbreeding. This pattern is consistent with recent accumulation of autozygosity despite the lower overall F_ROH_ compared with other Merino and Merino-derived breeds.

Inbreeding levels were not the same across different groups within the ASM population. Farm 1 showed higher F_ROH_ and F_HBD_ than Farm 2, and also a higher number of long ROH and HBD segments. Differences between sexes were less pronounced, but rams showed slightly higher average F_ROH_ and F_HBD_ than ewes and also a higher average number of segments longer than 16 Mb. This observation is relevant from a conservation perspective because breeding males typically constitute a smaller, more strongly selected subset of livestock populations. Unequal genetic contributions of sires can increase coancestry and accelerate the accumulation of inbreeding in subsequent generations, particularly in small or closed populations [6,8]. Similar sex-related differences have been reported in other livestock populations [35]. However, the higher inbreeding observed in ASM rams should be interpreted cautiously, because the number of analysed rams was small. This increases sampling uncertainty and makes the sex comparison less robust than the comparison between farms.

Inbreeding estimates based on HBD segments are generally considered more accurate than those derived from ROH when using 50k SNP data. This is particularly evident for short segments comprising a small number of SNPs, where the differences between methods can be substantial [36]. A similar pattern was observed in the ASM population, where major discrepancies between ROH and HBD estimates were detected for short segments (up to 4 Mb). Given these differences, both approaches were applied in this study. F_ROH_ was calculated to enable direct comparison with previously published inbreeding rates of Merino and Merino-derived breeds. At the same time, F_HBD_ was used as a more precise approach for assessing current inbreeding levels and for future monitoring.

Consistent with recent inbreeding, a low current Ne and a decline in the historical trajectory of Ne were observed. Although the three LD-based methods produced different contemporary Ne estimates, all values were relatively low. Differences among methods are not unexpected, because LD-based Ne estimation is sensitive to several factors, including sample size, allele-frequency filtering, physical linkage, mating structure, migration and population substructure [37,38]. In the ASM population, the presence of two farm-based subgroups and possible recent gene flow from introduced animals may have influenced linkage disequilibrium patterns and contributed to differences among software estimates. For this reason, the contemporary Ne values should be interpreted primarily as evidence of a limited effective size rather than as exact estimates of the number of breeding individuals. The historical Ne trajectory indicated a reduction in Ne towards the present, with the highest estimated value observed approximately nine generations ago. This pattern is consistent with the known demographic contraction of fine-wool sheep populations following the decline in the economic importance of wool production and with the current status of ASM as a small population maintained in only two farms. However, historical Ne estimates based on linkage disequilibrium should also be interpreted cautiously in the presence of population structure, admixture or recent gene flow, because these processes can generate LD patterns that partly mimic demographic change. Recent developments in GONE2 and currentNe2 explicitly address population structure and data quality as important factors in LD-based demographic inference [20]. Therefore, the estimated decline should be understood as a genomic signal consistent with recent demographic reduction, rather than as a precise reconstruction of population history.

In contrast to the observed decline in N_e_ and the high proportion of recent inbreeding, negative F_IS_ values and higher H_o_ than H_e_ were observed when individuals were evaluated separately by farms. When the ASM population was analysed as a single group, H_o_ and H_e_ were approximately equal, and F_IS_ was close to zero. The discrepancy between F_IS_ estimates obtained for the whole population and for farms can be attributed to population substructure. In subdivided populations, the mixing of genetically differentiated groups typically reduces observed heterozygosity relative to expectations, a phenomenon known as the Wahlund effect, which results in increased F_IS_ values in the metapopulation [39]. When individuals were grouped by sex, F_IS_ was negative in rams, whereas the value observed in ewes was close to zero and similar to that obtained for the whole population. This similarity is expected because ewes represented the vast majority of analysed individuals (477 of 491) and therefore largely determined the estimate obtained for the pooled population. In contrast, the negative F_IS_ observed in rams should be treated cautiously because only 14 rams were analysed, making this estimate considerably more sensitive to the composition of the small sample. The apparent discrepancy between F_ROH_ and F_HBD_, indicating recent inbreeding, and heterozygosity-based measures, indicating heterozygote excess in some groups, likely reflects the coexistence of different processes within the population. Substantial variability in inbreeding levels among individuals was observed (Figure 2B), ranging from highly inbred individuals (e.g., potentially reflecting recent close-relative mating) to individuals with near-zero F_ROH_ and F_HBD_, and even negative F_HOM_ (e.g., possible offspring of introduced rams). We assume that this heterogeneity is caused by simultaneous inbreeding and outbreeding and contrasting patterns observed between different indicators of genetic diversity. Moreover, this variability suggests that average inbreeding values alone are insufficient to describe the ASM population, because they may obscure the coexistence of animals with very different levels of autozygosity. Such variability in inbreeding could indicate a lack of a systematized mating scheme, which would minimize population inbreeding, avoid mating of closely related individuals and support the sustainability of unique AnGR [9].

### 4.2. Population Structure

Analysis of population structure using genome-wide SNP data demonstrated that ASM represents a genetically distinct group among the analysed Merino and Merino-derived breeds. ASM showed the lowest genetic differentiation from Australian Poll Merino, Australian Merino, Australian Industry Merino, Rambouillet, Spanish Merinos Estrema Dura and Chinese Merino. This pattern should be interpreted as evidence of a shared Merino-derived genetic background rather than direct recent gene flow between these populations.

The relatively close relationship with Rambouillet is consistent with the documented formation of Askanian Merino, in which American Rambouillet rams were used on local Merino ewes, with a small contribution of Précoce blood aimed at improving meat traits [3]. The low differentiation from Australian Merino-derived breeds and Spanish Merinos Estrema Dura is also logical. Australian Merino populations were formed from several Merino-related sources, including Spanish and French/Rambouillet stocks, while Spanish Merinos Estrema Dura represents a current commercial Spanish Merino population integrated within the broader Merino genetic cluster [2,11]. Therefore, the genomic position of ASM most likely reflects the usage of related fine-wool Merino and Rambouillet individuals during breed formation, rather than a simple relationship with a single ancestral source. The strongest differentiation from ASM was observed for European Mouflon, Macarthur Merino and Spanish Merinos Cordoba. The separation of European Mouflon was expected because it represents a wild sheep population rather than a domestic Merino-derived breed. The distinct position of Macarthur Merino and Spanish Merinos Cordoba may reflect their specific breed histories and stronger drift within these reference groups. However, these results should be interpreted cautiously because relatively small sample sizes represented both populations in the reference dataset, which may affect estimates of genetic distance and differentiation.

A clear internal subdivision of the Slovak ASM population was detected, indicating that the two farms represent partly distinct management units within the national population. It most likely reflects separate farm management, including limited exchange of breeding animals. Population subdivision is a common feature in livestock breeds, particularly under natural mating systems, where geographical distance can strongly limit gene flow [40,41]. The observed differentiation may also have been influenced by gene flow, because rams from the Ukrainian Askanian Merino population were introduced into Farm 1 to support genetic diversity. However, this contribution cannot be directly quantified without reference samples from the Ukrainian Askanian Merino population. Notably, the level of genetic differentiation observed between the two ASM subpopulations appears to be more pronounced than that observed between some Merino and Merino-derived breeds. In addition to the factors mentioned above, it cannot be excluded that this level of differentiation may have also been influenced by additional admixture of the ASM population in recent decades.

### 4.3. Genomics-Informed Mating Management

The results of genomic relatedness and GDI showed that ASM rams differed in their relationship to the current breeding population and in their potential value for maintaining genomic diversity. Average genomic relatedness of rams ranged from 0.034 to 0.150, while GDI ranged from 0.045 to 0.236. These differences are relevant for conservation management because the choice and use of breeding males can strongly influence the distribution of genetic contributions in subsequent generations. In small livestock populations, the objective is therefore not only to reduce the average relatedness of selected mating pairs, but also to avoid excessive contributions of a limited number of sires and to maintain genetic diversity across future generations [6,8].

To address this issue, an optimized mating allocation based on genomic relatedness was proposed. The objective was to minimize relatedness between mating pairs while respecting practical breeding constraints. The initial allocation showed that different ram-use constraints produced different levels of average genomic relatedness. The strategy based on equal use of rams resulted in the highest relatedness, whereas Max50 achieved the lowest value. This reflects the fact that equal use of all rams is not necessarily optimal when individual rams differ in their average relatedness to the available ewes. However, minimizing immediate relatedness alone does not automatically define the most suitable strategy, because practical implementation and the distribution of sire contributions must also be considered. The forward simulation showed that the immediate reduction in genomic relatedness between selected mating pairs translated into a slower accumulation of inbreeding across generations. In all optimized scenarios, inbreeding initially decreased below the founder level during the first three generations, whereas random mating scenarios showed a continuous increase from the beginning of the simulation. This initial decrease most likely reflected the use of the common sire pool together with the selection of less related ewe–ram combinations in the currently structured population. Although the Max50 scenario achieved the lowest average genomic relatedness in the initial allocation, this did not translate into a clearly better long-term outcome compared with the Min15_Max50 scenario. In the forward simulation, both Max50 optimized and Min15_Max50 optimized reached almost identical final levels of genomic inbreeding. The difference between these two scenarios is therefore mainly practical. The Max50 scenario allowed very uneven ram use, with some rams assigned to only 1–4 ewes. Although this is optimal from the perspective of minimizing pairwise relatedness in the current generation, it is difficult to implement under natural mating conditions. It may increase the risk that some rams do not contribute offspring in a given breeding season. In contrast, the Min15_Max50 scenario retained the same level of inbreeding while ensuring a minimum number of mating opportunities per used ram. Therefore, among the tested scenarios, Min15_Max50 optimized can be considered the most appropriate compromise between genetic optimization and practical feasibility.

The simulations also showed that mating allocation affected the genetic structure of the ASM population. The initial F_ST_ between farms decreased across all simulated scenarios, indicating that the use of a common sire pool increased genetic connectedness between the two farms. This result should not be interpreted as a recommendation to eliminate farm-related structure completely. Rather, it suggests that strict separation of the two farms would be unsuitable as a long-term strategy for such a small population, because it could further increase relatedness and inbreeding within each farm. At the same time, uncontrolled exchange of breeding animals would not be sufficient, because gene flow alone does not prevent matings between related individuals. Therefore, the ASM population should be managed as one small national population, while mating allocation should aim to minimize the accumulation of inbreeding. The proposed approach should not be interpreted as a complete breeding programme for ASM. Rather, it represents a genomics-informed improvement of mating allocation that can be implemented within the existing breeding structure. Its practical value lies in identifying ewe–ram combinations that reduce expected genomic relatedness, limit the accumulation of inbreeding and maintain more balanced use of available rams.

From a methodological point of view, the use of a burn-in phase is common in stochastic breeding simulations, particularly when the aim is to generate a common starting point for comparing alternative scenarios [42]. However, a burn-in phase was not applied in the present simulation because the aim was not to generate an artificial population at demographic or genetic equilibrium. Instead, the objective was to evaluate the expected consequences of mating allocation directly from the observed ASM population, including its current farm-related structure, genomic inbreeding distribution and relatedness among available breeding animals. Applying a conventional burn-in phase would have altered these initial conditions and reduced the relevance of the simulation for practical implementation in the current population. For this reason, generation 0 was defined as the observed active breeding population.

## 5. Conclusions

This study demonstrates the potential of integrating genomic information into conservation breeding management, moving beyond the assessment of genetic diversity towards its practical application in breeding decisions. In the Slovak Askanian Merino population, genomic analyses provided evidence of recent inbreeding and suggested an ongoing erosion of genetic diversity, highlighting the need for a systematic mating strategy to avoid matings between closely related individuals. In this context, the proposed mating approach offers a means of slowing the accumulation of inbreeding while respecting practical breeding constraints. However, effective conservation management also requires balancing the minimization of genomic relatedness with the appropriate use of available breeding males, as the genetically optimal mating allocation may not always be the most feasible under practical conditions. By combining genomic monitoring, optimized mating allocation and forward simulations, this study provides a flexible framework that could be adapted to other small and endangered livestock populations. Such an approach could support more informed breeding decisions and contribute to the long-term preservation of animal genetic resources.

## Figures and Tables

**Table 1:** List of Merino and Merino-derived breeds used in this study.

Breed	No. of Animals Before Quality Control	No. of Animals After Quality Control	Genotyping Platform	Source of Data
Slovak Askanian Merino (ASM)	502	491	GGP Ovine 50k	In-house
Australian Industry Merino (AIM)	88	88	OvineSNP50 BeadChip	[2,11]
Australian Merino (AUM)	50	50		
Australian Poll Merino (APM)	98	98		
Chinese Merino (CHM)	23	23		
European Mouflon (EUM)	24	24		
Macarthur Merino (MCM)	10	10		
Merinolandschaf (MLS)	21	21		
Rambouillet (RAM)	102	102		
Spanish Merinos Cordoba (MCO)	7	7		
Spanish Merinos Estrema Dura (MED)	13	13		

**Table 2:** Average values ± standard errors of genomic diversity indicators in the Slovak Askanian Merino population.

Group	No. of Individuals	H_o_	H_e_	F_IS_	F_HOM_	F_HBD_	F_ROH_
Farm1	118	0.396 ± 0.003	0.369 ± 0.001	−0.066 ± 0.001	0.041 ± 0.008	0.052 ± 0.006	0.045 ± 0.006
Farm2	373	0.416 ± 0.008	0.412 ± 0.001	−0.010 ± 0.000	−0.008 ± 0.002	0.017 ± 0.001	0.011 ± 0.001
Ewes	477	0.411 ± 0.001	0.413 ± 0.001	0.004 ± 0.000	0.004 ± 0.003	0.025 ± 0.002	0.019 ± 0.002
Rams	14	0.412 ± 0.011	0.357 ± 0.001	−0.144 ± 0.001	0.002 ± 0.027	0.031 ± 0.018	0.027 ± 0.017
All	491	0.411 ± 0.001	0.412 ± 0.001	0.004 ± 0.000	0.004 ± 0.003	0.025 ± 0.002	0.019 ± 0.002

H_o_—observed heterozygosity, H_e_—expected heterozygosity, F_IS_—Wright’s inbreeding coefficient, F_HOM_—inbreeding derived from the genome-wide homozygous excess, F_HBD_—inbreeding coefficient based on homozygous-by-descent segments longer than 1 Mb, F_ROH_—inbreeding coefficient based on runs of homozygosity longer than 1 Mb. Averages and standard errors for H_o_, H_e_ and F_IS_ were calculated across loci, whereas those for F_HOM_, F_HBD_ and F_ROH_ were calculated across individuals.

**Table 3:** Average number ± standard errors of ROH and HBD segments per individual across length categories in the Slovak Askanian Merino population.

	Average Number of Segments per Individual					
	Total	1–2 Mb	2–4 Mb	4–8 Mb	8–16 Mb	>16 Mb
ROH (PLINK)	5.866 ± 0.301	0.648 ± 0.039	1.768 ± 0.078	1.737 ± 0.089	0.866 ± 0.082	0.847 ± 0.120
HBD (RzooRoH)	13.971 ± 0.493	6.623 ± 0.227	3.662 ± 0.133	1.925 ± 0.102	0.888 ± 0.083	0.873 ± 0.121

**Table 4:** Genomic diversity index (GDI) and genomic relatedness of the 14 ASM rams, with average relatedness ± standard errors across pairwise comparisons with individuals in the analysed population.

Ram ID	GDI	Average Relatedness ± SE	Min. Relatedness	Max. Relatedness
A	0.236	0.034 ± 0.004	0	0.628
B	0.102	0.041 ± 0.004	0	0.538
C	0.045	0.042 ± 0.004	0	0.369
D	0.049	0.046 ± 0.004	0	0.516
E	0.046	0.047 ± 0.004	0	0.5
F	0.049	0.051 ± 0.004	0	0.685
G	0.063	0.054 ± 0.005	0	0.539
H	0.064	0.056 ± 0.005	0	0.5
I	0.068	0.061 ± 0.005	0	0.597
J	0.073	0.063 ± 0.006	0	0.584
K	0.074	0.066 ± 0.007	0	0.677
L	0.086	0.066 ± 0.005	0	0.512
M	0.096	0.078 ± 0.008	0	0.650
N	0.153	0.150 ± 0.005	0	0.558

## Data Availability

The SNP data of the Slovak Askanian Merino population are available from the corresponding author upon reasonable request. These data are not publicly available due to data-sharing restrictions. The comparative genotypic datasets used in this study are publicly available from the sources cited in the manuscript.

## Supplementary Materials

The following supporting information can be downloaded at https://www.mdpi.com/article/10.3390/ani16213413/s1. Supplementary Figure S1. StructureSelector evaluation of the optimal number of K based on the MedMedK, MedMeanK, MaxMedK and MaxMeanK criteria.; Supplementary Figure S2. Proportion of genetic admixture among Merino and Merino-derived breeds at K = 2–20. ASM—Slovak Askanian Merino, AIM—Australian Industry Merino, AUM—Australian Merino, APM—Australian Poll Merino, CHM—Chinese Merino, EUM—European Mouflon, MCM—Macarthur Merino, MLS—Merinolandschaf, RAM—Rambouillet, MCO—Spanish Merinos Cordoba, MED—Spanish Merinos Estrema Dura.; Supplementary Table S1. Average number (±SE) of HBD segments per individual across length categories in different groups of the ASM population.; Supplementary Table S2. Average number (±SE) of ROH segments per individual across length categories in different groups of the ASM population.; Supplementary Table S3. Average F_HBD_ calculated from homozygous-by-descent segments longer than 16 Mb across 15 simulation replicates. Values are presented as mean ± SE, with standard deviation (SD) and observed range (minimum–maximum) across replicates for each scenario and generation.; Supplementary Table S4. Average F_HBD_ calculated from homozygous-by-descent segments longer than 8 Mb across 15 simulation replicates. Values are presented as mean ± SE, with standard deviation (SD) and observed range (minimum–maximum) across replicates for each scenario and generation.; Supplementary Table S5. Average F_HBD_ calculated from homozygous-by-descent segments longer than 4 Mb across 15 simulation replicates. Values are presented as mean ± SE, with standard deviation (SD) and observed range (minimum–maximum) across replicates for each scenario and generation.; Supplementary Table S6. Average F_HBD_ calculated from homozygous-by-descent segments longer than 2 Mb across 15 simulation replicates. Values are presented as mean ± SE, with standard deviation (SD) and observed range (minimum–maximum) across replicates for each scenario and generation.; Supplementary Table S7. Average F_ST_ between farms across simulated mating scenarios and generations. Values are presented as the mean ± standard error (SE) across 15 simulation replicates. The standard deviation (SD) indicates between-replicate variability, and the observed range shows the minimum and maximum F_ST_ values across replicates for each scenario and generation.

## Abbreviations

The following abbreviations are used in this manuscript:

AnGR	Animal genetic resources
ASM	Slovak Askanian Merino
DAPC	Discriminant analysis of principal components
F_HBD_	Inbreeding coefficient based on homozygous-by-descent segments
F_HOM_	Inbreeding coefficient based on excess of homozygosity
F_IS_	Wright’s inbreeding coefficient
F_ROH_	Inbreeding coefficient based on runs of homozygosity
F_ST_	Fixation index
GDI	Genomic diversity index
HBD	Homozygous-by-descent
He	Expected heterozygosity
Ho	Observed heterozygosity
IBD	Identity by descent
IBS	Identity by state
LD	Linkage disequilibrium
Ne	Effective population size
PCoA	Principal coordinates analysis
ROH	Runs of homozygosity
SE	Standard error
SNP	Single-nucleotide polymorphism
