# Supplementary material for: Genomics-Informed Monitoring and Management of Genetic Diversity in a Small and Endangered Livestock Population: A Case Study of the Slovak Askanian Merino Sheep
Source: Animals (Basel). 2026 Oct 29;16(21):3413. doi: 10.3390/ani16213413 (PMC13761617; doi:10.3390/ani16213413)
Supplement: Supplementary file 1 [file animals-16-03413-s001.zip › animals-4600305-supplementary.pdf]

Genomics-informed monitoring and management of genetic diversity in a small and endangered livestock population: A case study of the Slovak Askanian Merino sheep

Adrián Halvoník[1], Nina Moravčíková[1], Ivan Pavlík[2], Ján Tomka[2], Luboš Vostrý[3], Hana Vostrá-Vydrová[3], Radovan Kasarda[1]

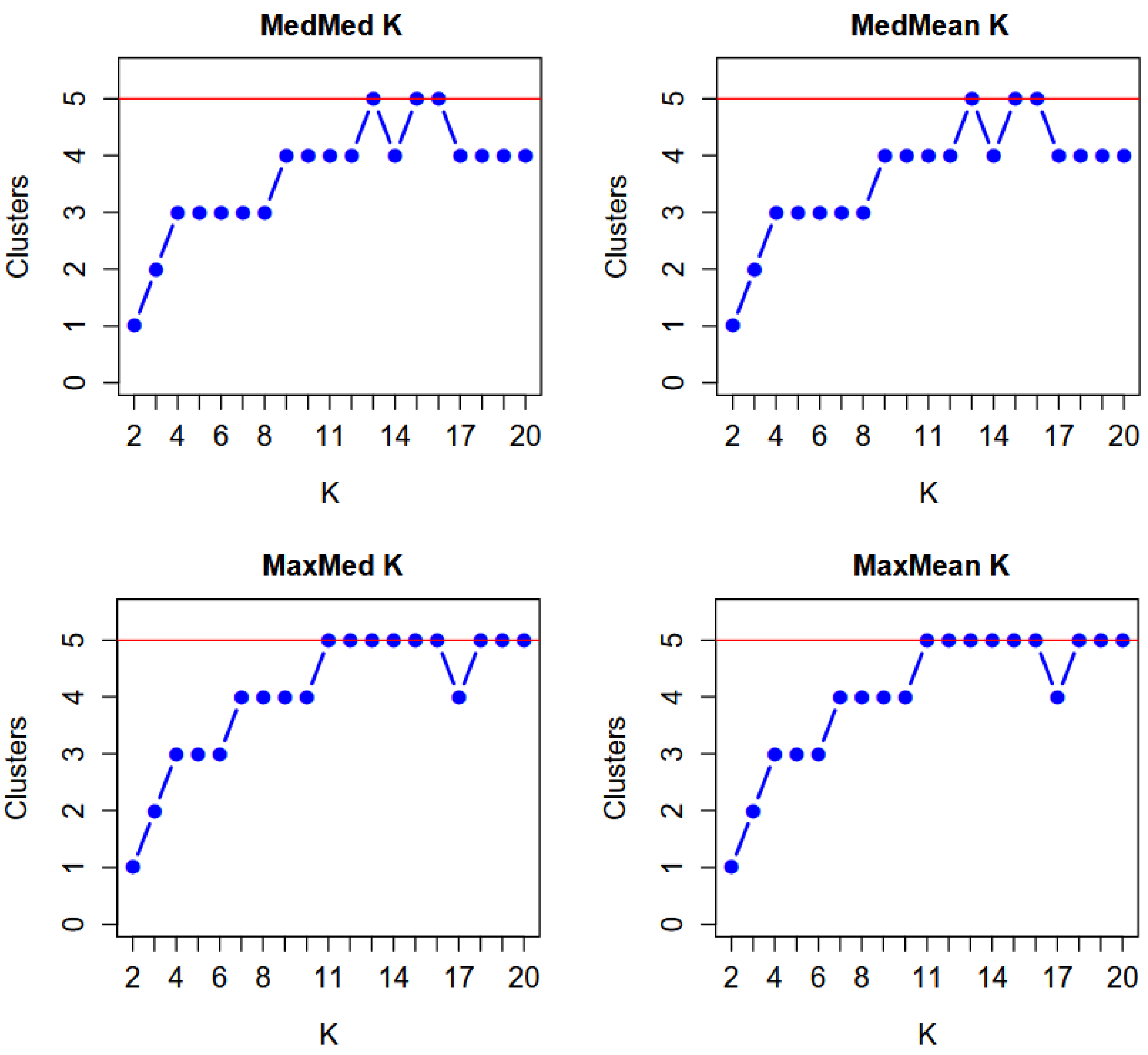

Supplementary Figure S1. StructureSelector evaluation of the optimal number of K based on the MedMedK, MedMeanK, MaxMedK and MaxMeanK criteria.

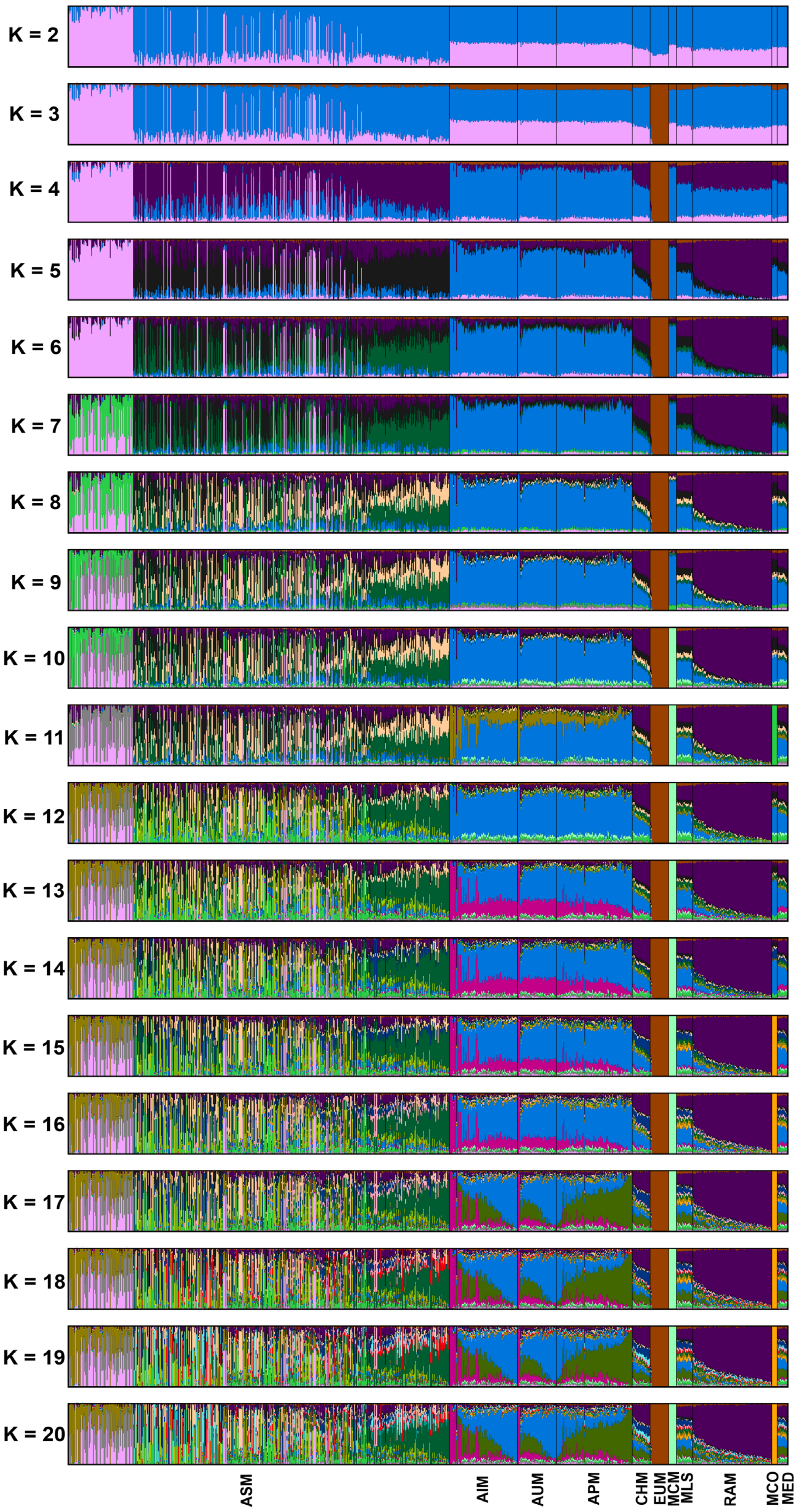

Supplementary Figure S2. Proportion of genetic admixture among Merino and Merino-derived breeds at K = 2–20. ASM – Slovak Askanian Merino, AIM - Australian Industry Merino, AUM - Australian Merino, APM – Australian Poll Merino, CHM – Chinese Merino, EUM – European Mouflon, MCM – Macarthur Merino, MLS – Merinolandschaf, RAM – Rambouillet, MCO – Spanish Merinos Cordoba, MED – Spanish Merinos Estrema Dura**.**

Supplementary Table S1. Average number (± SE) of HBD segments per individual across length categories in different groups of the ASM population.

Group	1–2 Mb	2–4 Mb	4–8 Mb	8–16 Mb	>16 Mb	Total
Rams	4.071 ± 1.202	2.643 ± 0.626	1.929 ± 0.597	0.714 ± 0.354	1.571 ± 1.166	10.929 ± 3.360
Ewes	6.698 ± 0.231	3.692 ± 0.136	1.925 ± 0.103	0.893 ± 0.085	0.853 ± 0.120	14.061 ± 0.498
Farm 1	6.085 ± 0.386	3.737 ± 0.267	2.831 ± 0.284	1.992 ± 0.266	2.551 ± 0.403	17.195 ± 1.346
Farm 2	6.794 ± 0.273	3.638 ± 0.154	1.638 ± 0.095	0.539 ± 0.060	0.343 ± 0.078	12.952 ± 0.480
All	6.623 ± 0.227	3.662 ± 0.133	1.925 ± 0.102	0.888 ± 0.083	0.874 ± 0.121	13.971 ± 0.493

Supplementary Table S2. Average number (± SE) of ROH segments per individual across length categories in different groups of the ASM population.

Group	1–2 Mb	2–4 Mb	4–8 Mb	8–16 Mb	>16 Mb	Total
Rams	0.143 ± 0.097	1.143 ± 0.312	2.000 ± 0.524	0.786 ± 0.395	1.571 ± 1.166	5.643 ± 2.170
Ewes	0.662 ± 0.040	1.786 ± 0.080	1.730 ± 0.090	0.868 ± 0.084	0.826 ± 0.119	5.872 ± 0.303
Farm 1	0.508 ± 0.068	1.695 ± 0.147	2.593 ± 0.237	1.898 ± 0.260	2.542 ± 0.403	9.237 ± 0.950
Farm 2	0.692 ± 0.047	1.791 ± 0.092	1.466 ± 0.086	0.539 ± 0.061	0.311 ± 0.075	4.799 ± 0.233
All	0.648 ± 0.039	1.768 ± 0.078	1.737 ± 0.089	0.866 ± 0.082	0.847 ± 0.120	5.866 ± 0.301

Supplementary Table S3. Average F_{HBD} calculated from homozygous-by-descent segments longer than 16 Mb across 15 simulation replicates. Values are presented as mean ± SE, with standard deviation (SD) and observed range (minimum–maximum) across replicates for each scenario and generation.

Generation	Balanced opt.	Balanced rand.	Max50 opt.	Max50 rand.	Min15-max50 opt.	Min15-max50 rand.
**1**	**0.008 ± 0.000**<br>SD 0.001; range 0.007–0.009	**0.011 ± 0.000**<br>SD 0.001; range 0.009–0.013	**0.007 ± 0.000**<br>SD 0.000; range 0.007–0.008	**0.012 ± 0.000**<br>SD 0.001; range 0.010–0.013	**0.008 ± 0.000**<br>SD 0.001; range 0.007–0.009	**0.012 ± 0.000**<br>SD 0.002; range 0.011–0.016
**2**	**0.007 ± 0.000**<br>SD 0.000; range 0.006–0.008	**0.015 ± 0.000**<br>SD 0.001; range 0.013–0.017	**0.007 ± 0.000**<br>SD 0.001; range 0.006–0.007	**0.016 ± 0.001**<br>SD 0.002; range 0.014–0.020	**0.007 ± 0.000**<br>SD 0.001; range 0.006–0.009	**0.016 ± 0.001**<br>SD 0.003; range 0.013–0.021
**3**	**0.006 ± 0.000**<br>SD 0.001; range 0.005–0.007	**0.020 ± 0.000**<br>SD 0.002; range 0.018–0.022	**0.006 ± 0.000**<br>SD 0.001; range 0.005–0.007	**0.020 ± 0.001**<br>SD 0.002; range 0.017–0.023	**0.007 ± 0.000**<br>SD 0.001; range 0.005–0.008	**0.020 ± 0.001**<br>SD 0.003; range 0.016–0.025
**4**	**0.007 ± 0.000**<br>SD 0.001; range 0.006–0.010	**0.025 ± 0.001**<br>SD 0.003; range 0.021–0.031	**0.007 ± 0.000**<br>SD 0.001; range 0.006–0.008	**0.025 ± 0.001**<br>SD 0.003; range 0.019–0.028	**0.008 ± 0.000**<br>SD 0.001; range 0.006–0.009	**0.025 ± 0.002**<br>SD 0.005; range 0.017–0.033
**5**	**0.009 ± 0.000**<br>SD 0.001; range 0.008–0.012	**0.028 ± 0.001**<br>SD 0.003; range 0.024–0.032	**0.009 ± 0.000**<br>SD 0.001; range 0.007–0.011	**0.028 ± 0.001**<br>SD 0.004; range 0.024–0.034	**0.009 ± 0.000**<br>SD 0.001; range 0.007–0.011	**0.029 ± 0.002**<br>SD 0.006; range 0.020–0.039
**6**	**0.011 ± 0.001**<br>SD 0.002; range 0.008–0.015	**0.031 ± 0.001**<br>SD 0.003; range 0.028–0.037	**0.011 ± 0.000**<br>SD 0.002; range 0.009–0.015	**0.031 ± 0.001**<br>SD 0.004; range 0.026–0.039	**0.011 ± 0.000**<br>SD 0.001; range 0.008–0.013	**0.031 ± 0.002**<br>SD 0.007; range 0.021–0.040
**7**	**0.013 ± 0.001**<br>SD 0.002; range 0.010–0.016	**0.032 ± 0.001**<br>SD 0.003; range 0.028–0.039	**0.012 ± 0.000**<br>SD 0.002; range 0.010–0.016	**0.034 ± 0.001**<br>SD 0.004; range 0.028–0.041	**0.012 ± 0.001**<br>SD 0.002; range 0.009–0.015	**0.034 ± 0.002**<br>SD 0.007; range 0.026–0.045
**8**	**0.015 ± 0.000**<br>SD 0.001; range 0.013–0.017	**0.034 ± 0.001**<br>SD 0.003; range 0.029–0.040	**0.014 ± 0.001**<br>SD 0.002; range 0.012–0.017	**0.036 ± 0.001**<br>SD 0.004; range 0.030–0.045	**0.013 ± 0.001**<br>SD 0.002; range 0.010–0.016	**0.036 ± 0.002**<br>SD 0.006; range 0.027–0.046
**9**	**0.016 ± 0.001**<br>SD 0.002; range 0.013–0.018	**0.037 ± 0.001**<br>SD 0.003; range 0.033–0.043	**0.015 ± 0.001**<br>SD 0.002; range 0.013–0.018	**0.038 ± 0.002**<br>SD 0.006; range 0.032–0.051	**0.015 ± 0.000**<br>SD 0.001; range 0.012–0.017	**0.038 ± 0.002**<br>SD 0.006; range 0.033–0.048
**10**	**0.017 ± 0.001**<br>SD 0.002; range 0.015–0.020	**0.039 ± 0.001**<br>SD 0.004; range 0.034–0.047	**0.017 ± 0.001**<br>SD 0.002; range 0.015–0.019	**0.040 ± 0.002**<br>SD 0.005; range 0.034–0.049	**0.016 ± 0.001**<br>SD 0.002; range 0.013–0.018	**0.040 ± 0.002**<br>SD 0.006; range 0.034–0.049

Supplementary Table S4. Average F_{HBD} calculated from homozygous-by-descent segments longer than 8 Mb across 15 simulation replicates. Values are presented as mean ± SE, with standard deviation (SD) and observed range (minimum–maximum) across replicates for each scenario and generation.

Generation	Balanced opt.	Balanced rand.	Max50 opt.	Max50 rand.	Min15-max50 opt.	Min15-max50 rand.
**1**	**0.011 ± 0.000**<br>SD 0.001; range 0.010–0.012	**0.015 ± 0.000**<br>SD 0.001; range 0.013–0.017	**0.011 ± 0.000**<br>SD 0.001; range 0.010–0.011	**0.016 ± 0.000**<br>SD 0.001; range 0.014–0.018	**0.011 ± 0.000**<br>SD 0.001; range 0.010–0.012	**0.016 ± 0.001**<br>SD 0.002; range 0.015–0.021
**2**	**0.010 ± 0.000**<br>SD 0.001; range 0.009–0.011	**0.021 ± 0.000**<br>SD 0.001; range 0.019–0.023	**0.010 ± 0.000**<br>SD 0.001; range 0.009–0.011	**0.022 ± 0.001**<br>SD 0.002; range 0.019–0.027	**0.011 ± 0.000**<br>SD 0.001; range 0.010–0.012	**0.022 ± 0.001**<br>SD 0.003; range 0.018–0.028
**3**	**0.010 ± 0.000**<br>SD 0.001; range 0.009–0.011	**0.028 ± 0.001**<br>SD 0.002; range 0.025–0.030	**0.010 ± 0.000**<br>SD 0.001; range 0.009–0.011	**0.027 ± 0.001**<br>SD 0.002; range 0.024–0.031	**0.010 ± 0.000**<br>SD 0.001; range 0.008–0.012	**0.028 ± 0.001**<br>SD 0.003; range 0.022–0.033
**4**	**0.012 ± 0.001**<br>SD 0.002; range 0.011–0.016	**0.035 ± 0.001**<br>SD 0.003; range 0.030–0.041	**0.012 ± 0.000**<br>SD 0.001; range 0.010–0.014	**0.035 ± 0.001**<br>SD 0.003; range 0.028–0.040	**0.013 ± 0.000**<br>SD 0.001; range 0.011–0.015	**0.035 ± 0.002**<br>SD 0.006; range 0.026–0.044
**5**	**0.016 ± 0.001**<br>SD 0.002; range 0.014–0.021	**0.040 ± 0.001**<br>SD 0.003; range 0.035–0.045	**0.016 ± 0.001**<br>SD 0.002; range 0.013–0.020	**0.040 ± 0.001**<br>SD 0.004; range 0.034–0.048	**0.016 ± 0.001**<br>SD 0.002; range 0.014–0.019	**0.041 ± 0.002**<br>SD 0.007; range 0.030–0.054
**6**	**0.020 ± 0.001**<br>SD 0.002; range 0.017–0.026	**0.044 ± 0.001**<br>SD 0.004; range 0.039–0.052	**0.020 ± 0.001**<br>SD 0.002; range 0.016–0.025	**0.045 ± 0.002**<br>SD 0.006; range 0.038–0.056	**0.020 ± 0.001**<br>SD 0.002; range 0.016–0.023	**0.045 ± 0.003**<br>SD 0.009; range 0.031–0.056
**7**	**0.024 ± 0.001**<br>SD 0.002; range 0.020–0.029	**0.047 ± 0.001**<br>SD 0.004; range 0.042–0.056	**0.023 ± 0.001**<br>SD 0.003; range 0.019–0.029	**0.049 ± 0.002**<br>SD 0.005; range 0.044–0.059	**0.023 ± 0.001**<br>SD 0.003; range 0.018–0.027	**0.049 ± 0.003**<br>SD 0.009; range 0.037–0.064
**8**	**0.028 ± 0.001**<br>SD 0.002; range 0.024–0.032	**0.050 ± 0.001**<br>SD 0.005; range 0.045–0.059	**0.026 ± 0.001**<br>SD 0.003; range 0.022–0.033	**0.053 ± 0.002**<br>SD 0.006; range 0.046–0.066	**0.026 ± 0.001**<br>SD 0.003; range 0.020–0.030	**0.053 ± 0.003**<br>SD 0.009; range 0.040–0.067
**9**	**0.031 ± 0.001**<br>SD 0.003; range 0.026–0.035	**0.054 ± 0.002**<br>SD 0.005; range 0.048–0.064	**0.029 ± 0.001**<br>SD 0.003; range 0.025–0.035	**0.057 ± 0.003**<br>SD 0.008; range 0.049–0.074	**0.028 ± 0.001**<br>SD 0.003; range 0.023–0.032	**0.057 ± 0.003**<br>SD 0.008; range 0.047–0.071
**10**	**0.034 ± 0.001**<br>SD 0.003; range 0.030–0.038	**0.058 ± 0.002**<br>SD 0.006; range 0.053–0.071	**0.032 ± 0.001**<br>SD 0.003; range 0.029–0.036	**0.061 ± 0.002**<br>SD 0.007; range 0.052–0.075	**0.032 ± 0.001**<br>SD 0.003; range 0.027–0.035	**0.060 ± 0.003**<br>SD 0.009; range 0.052–0.074

Supplementary Table S5. Average F_{HBD} calculated from homozygous-by-descent segments longer than 4 Mb across 15 simulation replicates. Values are presented as mean ± SE, with standard deviation (SD) and observed range (minimum–maximum) across replicates for each scenario and generation.

Generation	Balanced opt.	Balanced rand.	Max50 opt.	Max50 rand.	Min15-max50 opt.	Min15-max50 rand.
**1**	**0.014 ± 0.000**<br>SD 0.001; range 0.013–0.016	**0.019 ± 0.000**<br>SD 0.001; range 0.017–0.021	**0.014 ± 0.000**<br>SD 0.001; range 0.013–0.015	**0.020 ± 0.000**<br>SD 0.001; range 0.018–0.022	**0.014 ± 0.000**<br>SD 0.001; range 0.013–0.015	**0.020 ± 0.001**<br>SD 0.002; range 0.018–0.024
**2**	**0.013 ± 0.000**<br>SD 0.001; range 0.012–0.015	**0.026 ± 0.000**<br>SD 0.001; range 0.023–0.028	**0.013 ± 0.000**<br>SD 0.001; range 0.012–0.014	**0.026 ± 0.001**<br>SD 0.002; range 0.024–0.032	**0.014 ± 0.000**<br>SD 0.001; range 0.013–0.016	**0.027 ± 0.001**<br>SD 0.003; range 0.022–0.032
**3**	**0.013 ± 0.000**<br>SD 0.001; range 0.012–0.015	**0.033 ± 0.001**<br>SD 0.002; range 0.030–0.035	**0.013 ± 0.000**<br>SD 0.001; range 0.012–0.014	**0.032 ± 0.001**<br>SD 0.002; range 0.029–0.036	**0.014 ± 0.000**<br>SD 0.001; range 0.011–0.016	**0.033 ± 0.001**<br>SD 0.004; range 0.028–0.038
**4**	**0.017 ± 0.001**<br>SD 0.002; range 0.015–0.020	**0.041 ± 0.001**<br>SD 0.003; range 0.036–0.047	**0.016 ± 0.001**<br>SD 0.002; range 0.013–0.019	**0.040 ± 0.001**<br>SD 0.003; range 0.034–0.046	**0.017 ± 0.000**<br>SD 0.001; range 0.015–0.020	**0.041 ± 0.002**<br>SD 0.006; range 0.032–0.051
**5**	**0.022 ± 0.001**<br>SD 0.002; range 0.019–0.027	**0.047 ± 0.001**<br>SD 0.003; range 0.042–0.051	**0.021 ± 0.001**<br>SD 0.002; range 0.018–0.025	**0.047 ± 0.001**<br>SD 0.005; range 0.040–0.056	**0.022 ± 0.001**<br>SD 0.002; range 0.019–0.025	**0.048 ± 0.002**<br>SD 0.007; range 0.037–0.062
**6**	**0.026 ± 0.001**<br>SD 0.003; range 0.023–0.033	**0.052 ± 0.001**<br>SD 0.004; range 0.047–0.060	**0.026 ± 0.001**<br>SD 0.003; range 0.021–0.033	**0.052 ± 0.002**<br>SD 0.006; range 0.045–0.064	**0.026 ± 0.001**<br>SD 0.002; range 0.023–0.030	**0.052 ± 0.003**<br>SD 0.009; range 0.038–0.066
**7**	**0.031 ± 0.001**<br>SD 0.003; range 0.027–0.037	**0.055 ± 0.001**<br>SD 0.005; range 0.050–0.065	**0.030 ± 0.001**<br>SD 0.003; range 0.025–0.038	**0.058 ± 0.002**<br>SD 0.006; range 0.053–0.068	**0.031 ± 0.001**<br>SD 0.003; range 0.026–0.035	**0.058 ± 0.003**<br>SD 0.010; range 0.045–0.075
**8**	**0.036 ± 0.001**<br>SD 0.003; range 0.032–0.042	**0.059 ± 0.002**<br>SD 0.005; range 0.054–0.069	**0.034 ± 0.001**<br>SD 0.004; range 0.028–0.043	**0.063 ± 0.002**<br>SD 0.007; range 0.055–0.076	**0.034 ± 0.001**<br>SD 0.003; range 0.028–0.039	**0.063 ± 0.003**<br>SD 0.010; range 0.048–0.078
**9**	**0.040 ± 0.001**<br>SD 0.003; range 0.035–0.045	**0.064 ± 0.002**<br>SD 0.005; range 0.058–0.076	**0.038 ± 0.001**<br>SD 0.003; range 0.033–0.046	**0.068 ± 0.003**<br>SD 0.009; range 0.058–0.086	**0.038 ± 0.001**<br>SD 0.003; range 0.032–0.042	**0.068 ± 0.003**<br>SD 0.009; range 0.056–0.083
**10**	**0.044 ± 0.001**<br>SD 0.003; range 0.040–0.049	**0.069 ± 0.002**<br>SD 0.006; range 0.064–0.084	**0.042 ± 0.001**<br>SD 0.003; range 0.038–0.048	**0.072 ± 0.003**<br>SD 0.008; range 0.063–0.089	**0.042 ± 0.001**<br>SD 0.003; range 0.037–0.046	**0.072 ± 0.003**<br>SD 0.010; range 0.062–0.087

Supplementary Table S6. Average F_{HBD} calculated from homozygous-by-descent segments longer than 2 Mb across 15 simulation replicates. Values are presented as mean ± SE, with standard deviation (SD) and observed range (minimum–maximum) across replicates for each scenario and generation.

Generation	Balanced opt.	Balanced rand.	Max50 opt.	Max50 rand.	Min15-max50 opt.	Min15-max50 rand.
**1**	**0.016 ± 0.000** SD 0.001; range 0.014–0.017	**0.021 ± 0.000** SD 0.001; range 0.019–0.023	**0.016 ± 0.000** SD 0.001; range 0.015–0.017	**0.022 ± 0.000** SD 0.001; range 0.020–0.023	**0.016 ± 0.000** SD 0.001; range 0.015–0.017	**0.022 ± 0.001** SD 0.002; range 0.020–0.026
**2**	**0.015 ± 0.000** SD 0.001; range 0.014–0.016	**0.028 ± 0.000** SD 0.001; range 0.025–0.030	**0.015 ± 0.000** SD 0.001; range 0.013–0.016	**0.028 ± 0.001** SD 0.002; range 0.025–0.034	**0.015 ± 0.000** SD 0.001; range 0.014–0.017	**0.028 ± 0.001** SD 0.004; range 0.024–0.034
**3**	**0.015 ± 0.000** SD 0.001; range 0.014–0.016	**0.035 ± 0.001** SD 0.002; range 0.032–0.038	**0.015 ± 0.000** SD 0.001; range 0.013–0.016	**0.034 ± 0.001** SD 0.002; range 0.031–0.038	**0.015 ± 0.000** SD 0.002; range 0.013–0.017	**0.035 ± 0.001** SD 0.004; range 0.030–0.040
**4**	**0.018 ± 0.001** SD 0.002; range 0.017–0.022	**0.043 ± 0.001** SD 0.003; range 0.038–0.050	**0.018 ± 0.001** SD 0.002; range 0.015–0.021	**0.043 ± 0.001** SD 0.003; range 0.036–0.048	**0.019 ± 0.000** SD 0.001; range 0.017–0.022	**0.043 ± 0.002** SD 0.006; range 0.035–0.053
**5**	**0.024 ± 0.001** SD 0.002; range 0.021–0.029	**0.049 ± 0.001** SD 0.003; range 0.045–0.053	**0.023 ± 0.001** SD 0.002; range 0.020–0.027	**0.049 ± 0.001** SD 0.005; range 0.043–0.058	**0.024 ± 0.001** SD 0.002; range 0.021–0.027	**0.050 ± 0.002** SD 0.007; range 0.039–0.064
**6**	**0.029 ± 0.001** SD 0.003; range 0.025–0.035	**0.055 ± 0.001** SD 0.004; range 0.049–0.062	**0.028 ± 0.001** SD 0.003; range 0.024–0.035	**0.055 ± 0.002** SD 0.006; range 0.047–0.066	**0.029 ± 0.001** SD 0.002; range 0.026–0.033	**0.055 ± 0.003** SD 0.009; range 0.040–0.069
**7**	**0.034 ± 0.001** SD 0.003; range 0.030–0.040	**0.058 ± 0.001** SD 0.005; range 0.053–0.068	**0.033 ± 0.001** SD 0.003; range 0.027–0.040	**0.061 ± 0.002** SD 0.006; range 0.055–0.071	**0.033 ± 0.001** SD 0.003; range 0.028–0.038	**0.061 ± 0.003** SD 0.010; range 0.048–0.078
**8**	**0.039 ± 0.001** SD 0.003; range 0.036–0.045	**0.062 ± 0.002** SD 0.005; range 0.056–0.073	**0.037 ± 0.001** SD 0.004; range 0.031–0.046	**0.066 ± 0.002** SD 0.007; range 0.058–0.080	**0.037 ± 0.001** SD 0.003; range 0.031–0.042	**0.066 ± 0.003** SD 0.010; range 0.051–0.082
**9**	**0.043 ± 0.001** SD 0.003; range 0.039–0.049	**0.067 ± 0.002** SD 0.005; range 0.061–0.079	**0.041 ± 0.001** SD 0.004; range 0.036–0.049	**0.071 ± 0.003** SD 0.009; range 0.060–0.090	**0.041 ± 0.001** SD 0.004; range 0.035–0.046	**0.071 ± 0.003** SD 0.009; range 0.060–0.086
**10**	**0.048 ± 0.001** SD 0.003; range 0.043–0.053	**0.073 ± 0.002** SD 0.007; range 0.067–0.088	**0.045 ± 0.001** SD 0.003; range 0.041–0.052	**0.076 ± 0.003** SD 0.008; range 0.066–0.093	**0.045 ± 0.001** SD 0.004; range 0.040–0.050	**0.075 ± 0.003** SD 0.010; range 0.065–0.091

Supplementary Table S7. Average F_{ST} between farms across simulated mating scenarios and generations. Values are presented as the mean ± standard error (SE) across 15 simulation replicates. The standard deviation (SD) indicates between-replicate variability, and the observed range shows the minimum and maximum F_{ST} values across replicates for each scenario and generation.

Generation	Balanced opt.	Balanced rand.	Max50 opt.	Max50 rand.	Min15-max50 opt.	Min15-max50 rand.
**1**	**0.049 ± 0.000**<br>SD 0.001; range 0.046–0.051	**0.051 ± 0.001**<br>SD 0.002; range 0.048–0.054	**0.050 ± 0.000**<br>SD 0.001; range 0.047–0.052	**0.052 ± 0.001**<br>SD 0.002; range 0.048–0.055	**0.048 ± 0.000**<br>SD 0.001; range 0.046–0.050	**0.051 ± 0.001**<br>SD 0.003; range 0.046–0.056
**2**	**0.028 ± 0.000**<br>SD 0.002; range 0.025–0.030	**0.037 ± 0.001**<br>SD 0.004; range 0.029–0.043	**0.029 ± 0.001**<br>SD 0.002; range 0.026–0.033	**0.037 ± 0.002**<br>SD 0.005; range 0.030–0.048	**0.029 ± 0.001**<br>SD 0.002; range 0.025–0.032	**0.036 ± 0.002**<br>SD 0.006; range 0.027–0.048
**3**	**0.017 ± 0.000**<br>SD 0.002; range 0.015–0.020	**0.028 ± 0.002**<br>SD 0.005; range 0.020–0.034	**0.018 ± 0.001**<br>SD 0.002; range 0.015–0.022	**0.029 ± 0.002**<br>SD 0.006; range 0.019–0.040	**0.018 ± 0.001**<br>SD 0.002; range 0.014–0.021	**0.025 ± 0.001**<br>SD 0.004; range 0.018–0.032
**4**	**0.012 ± 0.001**<br>SD 0.002; range 0.009–0.015	**0.021 ± 0.001**<br>SD 0.005; range 0.017–0.029	**0.014 ± 0.001**<br>SD 0.002; range 0.010–0.018	**0.023 ± 0.002**<br>SD 0.005; range 0.015–0.031	**0.013 ± 0.001**<br>SD 0.002; range 0.010–0.017	**0.018 ± 0.001**<br>SD 0.003; range 0.014–0.023
**5**	**0.012 ± 0.001**<br>SD 0.002; range 0.007–0.016	**0.018 ± 0.002**<br>SD 0.005; range 0.012–0.028	**0.014 ± 0.001**<br>SD 0.002; range 0.012–0.017	**0.019 ± 0.002**<br>SD 0.006; range 0.011–0.030	**0.013 ± 0.001**<br>SD 0.003; range 0.010–0.020	**0.018 ± 0.001**<br>SD 0.004; range 0.012–0.026
**6**	**0.013 ± 0.001**<br>SD 0.003; range 0.007–0.016	**0.016 ± 0.001**<br>SD 0.004; range 0.011–0.022	**0.014 ± 0.001**<br>SD 0.003; range 0.010–0.019	**0.018 ± 0.002**<br>SD 0.005; range 0.011–0.028	**0.012 ± 0.001**<br>SD 0.003; range 0.008–0.016	**0.015 ± 0.001**<br>SD 0.004; range 0.011–0.024
**7**	**0.011 ± 0.001**<br>SD 0.003; range 0.009–0.017	**0.015 ± 0.001**<br>SD 0.003; range 0.011–0.020	**0.014 ± 0.000**<br>SD 0.001; range 0.012–0.016	**0.017 ± 0.002**<br>SD 0.005; range 0.012–0.024	**0.012 ± 0.001**<br>SD 0.002; range 0.008–0.015	**0.015 ± 0.001**<br>SD 0.002; range 0.011–0.018
**8**	**0.011 ± 0.001**<br>SD 0.002; range 0.007–0.015	**0.013 ± 0.001**<br>SD 0.004; range 0.009–0.021	**0.015 ± 0.001**<br>SD 0.003; range 0.012–0.023	**0.016 ± 0.002**<br>SD 0.005; range 0.010–0.025	**0.012 ± 0.001**<br>SD 0.003; range 0.009–0.016	**0.014 ± 0.001**<br>SD 0.002; range 0.011–0.018
**9**	**0.011 ± 0.000**<br>SD 0.001; range 0.010–0.013	**0.014 ± 0.001**<br>SD 0.003; range 0.009–0.018	**0.014 ± 0.001**<br>SD 0.002; range 0.011–0.018	**0.017 ± 0.001**<br>SD 0.004; range 0.011–0.022	**0.013 ± 0.001**<br>SD 0.003; range 0.009–0.020	**0.014 ± 0.001**<br>SD 0.004; range 0.009–0.022
**10**	**0.011 ± 0.000**<br>SD 0.001; range 0.009–0.013	**0.013 ± 0.001**<br>SD 0.004; range 0.009–0.018	**0.012 ± 0.001**<br>SD 0.002; range 0.009–0.016	**0.016 ± 0.001**<br>SD 0.004; range 0.013–0.026	**0.014 ± 0.001**<br>SD 0.002; range 0.011–0.017	**0.015 ± 0.001**<br>SD 0.003; range 0.012–0.024
